# Conformity in mate choice, the overlooked social component of animal and human culture

**DOI:** 10.1111/brv.12899

**Published:** 2022-09-29

**Authors:** Sabine Nöbel, Antoine Jacquet, Guillaume Isabel, Arnaud Pocheville, Paul Seabright, Etienne Danchin

**Affiliations:** ^1^ Institute for Advanced Study in Toulouse (IAST), Université Toulouse 1 Capitole Toulouse France; ^2^ Laboratoire Évolution et Diversité Biologique (EDB UMR 5174), Université de Toulouse, CNRS, IRD 118 route de Narbonne F‐31062 Toulouse cedex 9 France; ^3^ Toulouse School of Economics (TSE), Université Toulouse 1 Capitole Toulouse France; ^4^ Centre de Recherches sur la Cognition Animale, Centre de Biologie Intégrative, Université de Toulouse, CNRS, UPS 118 route de Narbonne F‐31062 Toulouse cedex 9 France

**Keywords:** cultural evolution, conformity in humans, conformity in animals, sexual selection, mate choice, Fisher runaway process

## Abstract

Although conformity as a major driver for human cultural evolution is a well‐accepted and intensely studied phenomenon, its importance for non‐human animal culture has been largely overlooked until recently. This limited for decades the possibility of studying the roots of human culture. Here, we provide a historical review of the study of conformity in both humans and non‐human animals. We identify gaps in knowledge and propose an evolutionary route towards the sophisticated cultural processes that characterize humanity. A landmark in the study of conformity is Solomon Asch's famous experiment on humans in 1955. By contrast, interest in conformity among evolutionary biologists has only become salient since the turn of the new millennium. A striking result of our review is that, although studies of conformity have examined many biological contexts, only one looked at mate choice. This is surprising because mate choice is probably the only context in which conformity has self‐reinforcing advantages across generations. Within a metapopulation, i.e. a group of subpopulations connected by dispersing individuals, dispersers able to conform to the local preference for a given type of mate have a strong and multigenerational fitness advantage. This is because once females within one subpopulation locally show a bias for one type of males, immigrant females who do not conform to the local trend have sons, grandsons, etc. of the non‐preferred phenotype, which negatively and cumulatively affects fitness over generations in a process reminiscent of the Fisher runaway process. This led us to suggest a sex‐driven origin of conformity, indicating a possible evolutionary route towards animal and human culture that is rooted in the basic, and thus ancient, social constraints acting on mating preferences within a metapopulation. In a generic model, we show that dispersal among subpopulations within a metapopulation can effectively maintain independent Fisher runaway processes within subpopulations, while favouring the evolution of social learning and conformity at the metapopulation scale; both being essential for the evolution of long‐lasting local traditions. The proposed evolutionary route to social learning and conformity casts surprising light on one of the major processes that much later participated in making us human. We further highlight several research avenues to define the spectrum of conformity better, and to account for its complexity. Future studies of conformity should incorporate experimental manipulation of group majority. We also encourage the study of potential links between conformity and mate copying, animal aggregations, and collective actions. Moreover, validation of the sex‐driven origin of conformity will rest on the capacity of human and evolutionary sciences to investigate jointly the origin of social learning and conformity. This constitutes a stimulating common agenda and militates for a rapprochement between these two currently largely independent research areas.

## I. INTRODUCTION

Social learning is a form of learning that specifically uses information obtained from conspecifics or other animals or the products of their activities to gain information about the environment (adapted from Heyes, 1994). It has been recognized as a crucial learning process for the transmission and spread of adaptive behaviours within and among populations (Laland, 2004; Rendell *et al*., 2011; Aplin *et al*., 2015*b*). As such, social learning is the cornerstone of cultural traditions, since culture relies by definition on socially transmitted information (Boyd & Richerson, 2005).

A major form of social learning that is essential for the establishment of cultural traditions and social norms in humans is conformity (Boyd & Richerson, 1985; Henrich & Boyd, 1998). Several definitions of conformity (or conformist bias) have been used in the literature, depending on the field. They range from ‘copy the majority’, which is a simple positive frequency‐dependent bias in learning (e.g. Laland, 2004) to ‘Aschian conformity’, where personal knowledge or preference is overridden by countervailing options performed by others (Asch, 1955). Perhaps the most popular and widespread definition of conformist bias or conformist transmission is defined as the *disproportionately likely* adoption of the most common variant within the local population (Boyd & Richerson, 1985). Conformity allows individuals easily to grasp local behaviours and norms. In turn, it can generate socially learned traditions that are both resistant to erosion and robust to invasion of alternative variants, potentially persisting over generations. Thus, conformity can produce a combination of within‐group homogeneity and between‐group heterogeneity (see discussion in Section III).

Because of its central role for the emergence and maintenance of culture, conformity has long been a major topic of research in human sciences (Boyd & Richerson, 1985; Banerjee, 1992; Bernheim, 1994; Aoki, Lehmann & Feldman, 2011; Merrell, 2011; Claidière & Whiten, 2012; Efferson *et al*., 2016; Denton *et al*., 2020). By contrast, the study of conformity in non‐human animals became a major topic in evolutionary sciences only recently (e.g. Whiten, Horner & de Waal, 2005; Aplin *et al*., 2015*b*; van Leeuwen *et al*., 2015; Danchin *et al*., 2018), and a synthesis between these two scientific domains is still lacking. Here, we review the literature on conformity in both humans and non‐human animals, thus drawing a link between these two sides of the literature. This review allows us to identify gaps in knowledge and suggest a general model for the evolution of social learning and conformity. Our review further suggests that conformity in mate choice deserves closer attention as sexual selection is a strong evolutionary force that has been largely overlooked up to now in this context. We aim to inspire new research agendas in both human and non‐human animals to determine in which context, e.g. mate choice or foraging, the cognitive processes that underpin the evolution of animal and human culture could have evolved and how they interact.

## II. EARLY DEFINITIONS: CONFORMITY OR POSITIVE FREQUENCY‐DEPENDENT LEARNING?

In this section, we propose a brief history of the term ‘conformity’ pre‐dating Boyd & Richerson's (1985) definition of conformist transmission bias. Most uses of the term ‘conformity’ over this period would now be more accurately described as positive frequency‐dependent social learning [see Efferson *et al*. (2008) for a discussion around existing definitions of conformity].

The influence of groups on individual behaviour first attracted attention in the early twentieth century (Jenness, 1932; Sherif, 1935), but it was Asch (1955) who popularized the term *conformity* itself. Using a simple ‘visual judgement’ task, Asch documented that people were willing to abandon their own personal preferences (or convictions) when confronted with a disagreeing majority opinion. He ascribed this behaviour to social pressure and called this innate tendency to self‐align on group opinion ‘conformity’. Asch's findings became momentous in developing the field of social psychology. They were replicated many times across different age classes and cultures, even when factors such as group size, motivation, task difficulty or relevance were varied (Baron, Vandello & Brunsman, 1996; Bond, 2005; Bond & Smith, 1996; Griskevicius *et al*., 2006). Later, Latané's (1981) work on ‘social impact theory’ provided a tentative conceptual framework for these findings. Drawing a parallel with physics, Latané pictured social influence as a force emanating from the group, and acting on individuals (e.g. peer pressure). The intensity of this force could depend on the source group's size, its behavioural composition (the proportion of individuals performing the various behaviours), its status (such as prestige), or its proximity in time and space to the target individual (see also Latané & Wolf, 1981). Overall, social psychology had suggested a proximate reason for ‘conforming’, namely that people inherently dislike going against the majority.

Note that these early studies equated conformity with a linear increase in the likelihood of adopting a behaviour as that behaviour becomes more common in the population – what today is called positive frequency‐dependent social learning. Conformist transmission bias as defined by Boyd & Richerson (1985) is a particular case of this class of learning rules (adoption of the most common variant needs to be disproportionately likely). Still, debates from this early period may provide useful insights regarding positive frequency‐dependent social learning, and therefore, regarding conformist transmission bias in the Boyd & Richerson sense.

One such example is the question of whether positive frequency‐dependent social learning arises because of normative or informational reasons. In Asch's studies, following the majority was a response to peer pressure, but this does not have to be the case. Early on, Deutsch & Gerard (1955) remarked that social psychology had focused mainly on a single side of ‘conformity’ (here again understood as positive frequency‐dependent social learning), namely, the normative side. In what they called normative social influence (later also referred to as *social* conformity; e.g. Coleman, 2004), the commonest behaviour defines a social norm. If the norm is explicitly enforced, for instance by rewarding compliers and punishing non‐compliers, then individuals might adopt the normative behaviour through ordinary cognitive mechanisms for avoiding social and environmental risks; in this case, conforming simply means avoiding punishment. But individuals might also adopt the norm not because they consciously try to avoid social risks, but because of specific preferences for conforming, which might have proved adaptive over long timescales. In other words, the drive to conform could be an individually optimized response to social incentives, but it could also be the result of evolved, wired‐in preferences. For now, the literature remains unclear on whether normative conformity includes both these mechanisms, or just the latter (conforming because of an evolved, intrinsic drive to do so, e.g. self‐esteem concerns).

But Deutsch & Gerard (1955) described another driver of majority‐following: informational social influence (sometimes also called *instrumental* conformity; *cf*. Burdett *et al*., 2016). In this case, the behaviour of the majority does not define a norm, but instead reveals useful information about the environment. Take the example of durian (*Durio zibethinus*), a tropical fruit with a distinctly unpleasant smell which is widely popular in South‐east Asia. The naïve tourist might be willing to try it despite the stench, not for fear of social sanctions if he or she does not, but rather because he or she can *infer* from observing the majority behaviour that the fruit is actually palatable. As pointed out by Cialdini & Goldstein (2004) however, informational and normative conformity are actually interrelated – for instance, from these definitions it is unclear whether an individual using social information to navigate a social environment (e.g. to avoid punishment) is an example of normative or informational conformity.

Economists summarized the mechanisms and limitations of the informational channel in a famous decision problem. Suppose that, on a night out and looking for a good dinner, you stumble upon two adjacent restaurants. One is crowded, the other almost empty. Which restaurant will you choose? First, you should realize that the behaviour of others probably conveys information about quality. The safest choice is therefore to follow the ‘wisdom of the crowd’ and go for the crowded restaurant (i.e. conform). However, sometimes the crowd is wrong, as Banerjee (1992) and Bikhchandani, Hirshleifer & Welch (1992) pointed out. Indeed, in this problem, the first few customers are pivotal in determining group behaviour: if they choose the lower‐quality restaurant, all subsequent customers will copy their mistake. Such *informational cascades* were later observed in laboratory experiments with human subjects (Anderson & Holt, 1997). What these studies emphasized is the fragility of mass behaviour when it is driven by conformity, in the sense that it is susceptible to shifts when accurate information is publicly released. Back to the restaurant example, it means that if customers had had access to the restaurants' ratings on Yelp or Tripadvisor, then they could have ignored the mistake of the first few customers and chosen the best restaurant instead despite it being empty.

Economists also studied the normative channel. For instance, Bernheim (1994) considered a population in which individuals must balance their self‐interest with social esteem. Social esteem is obtained by adhering to a group norm, but individuals vary in how their self‐interest aligns with this norm. Bernheim (1994) showed that individuals whose self‐interest closely aligns with the norm will conform to it in order to reap the social esteem benefits. On the other hand, individuals whose self‐interest departs a lot from the norm will ignore that norm, but by doing so their behaviour flags them to the rest of society as undeserving of social esteem. This model illustrates how reward and punishment can enforce conformity around a social norm, even in the presence of individual heterogeneity. In a similar spirit, Kuran & Sandholm (2008) studied how individuals with heterogeneous preferences behave in the presence of coordination incentives; they also found that individuals tend to align their behaviour with the social norm.

This informational *versus* normative dichotomy, together with the above studies by economists, provides a convenient analytical structure for evolutionary biologists. They suggest that any tendency towards positive frequency‐dependent social learning should be rooted in one of these two channels, i.e. that it should have normative or informational benefits. However, informational and normative conformity are interrelated (David & Turner, 1996), and as such they are difficult to disentangle theoretically and empirically. This is reflected in the research on individual differences in conformity. For example, DeYoung, Peterson & Higgins (2002) looked at two personality types, namely *Stability* (emotional stability, agreeableness and conscientiousness) and *Plasticity* (extraversion, openness), and found that Stability is a positive predictor of conformity while Plasticity was negatively correlated with conformity. Thus, strong conformists tend to be very stable but also less able to adjust to novelty or change. This also suggests that positive frequency‐dependent social learning is not always the right answer to an environmental challenge, as we will see more in detail below. Finally, as a particular case of positive frequency‐dependent social learning, conformist transmission in the Boyd & Richerson sense should also obey these insights.

## III. CONFORMITY EVOLVES: A BENEFICIAL BIAS?

**Fig. 1:**
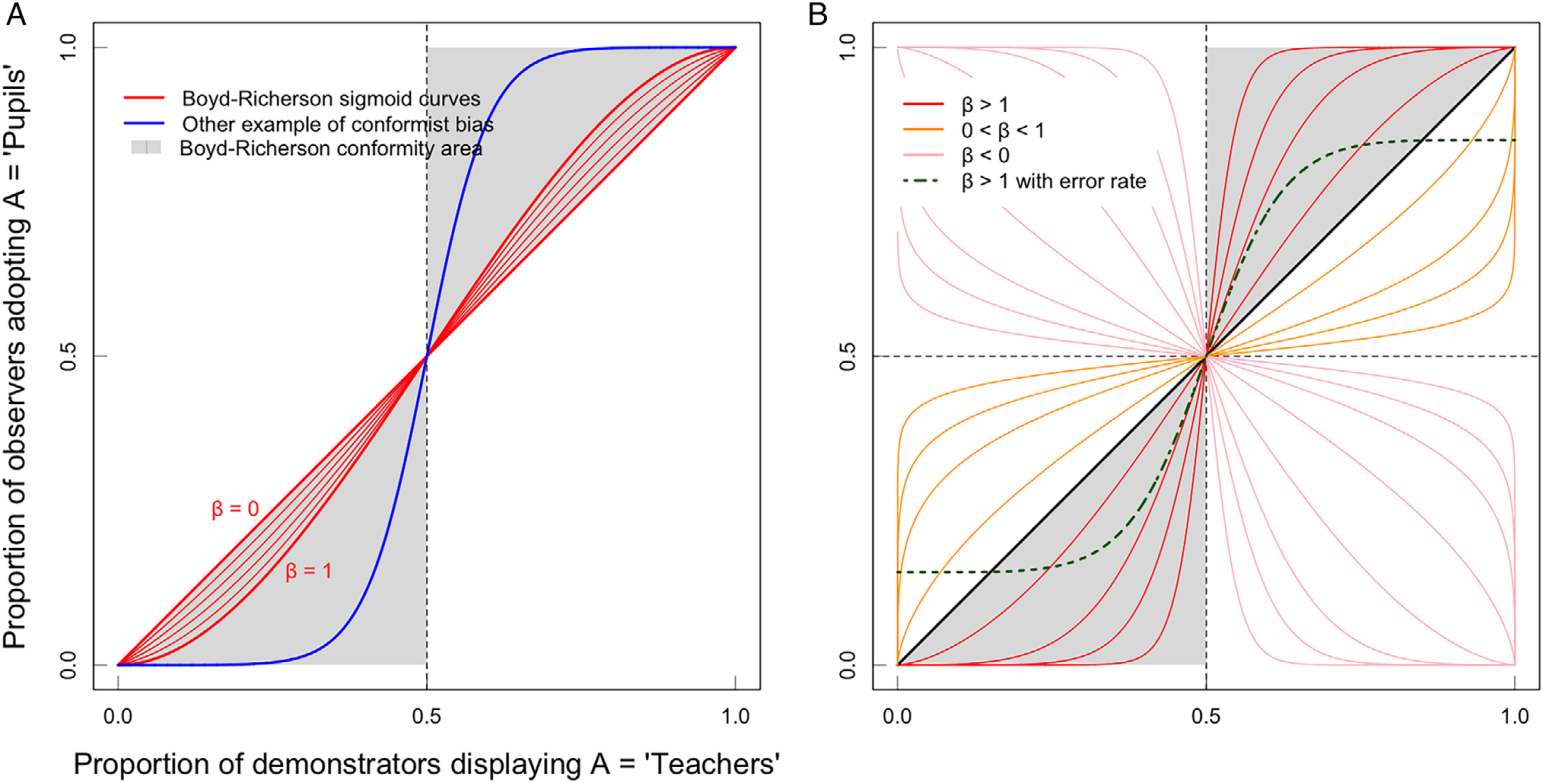
An alternative functional form for conformity. Both graphs depict the relationship between the proportion of demonstrators adopting one option (*x*‐axis) and the probability that an observer subsequently adopts that option (*y*‐axis; in a case with two options). On the left (A) we show Boyd & Richerson's (1985) definition of conformist bias. The grey areas correspond to conformity: observers are *disproportionately likely* to adopt the majority behaviour, i.e. more than under random matching. The red lines correspond to Boyd & Richerson's (1985) mathematical definition of conformist bias: they only cover a small part of the area designated for conformity. The blue line corresponds to a conformist learning rule that cannot be approached by Boyd & Richerson's (1985) definition. On the right (B) we show learning rules associated with the logistic expression from Section III.4. This expression is flexible enough to cover Boyd & Richerson's conformist bias or ‘hyper‐conformity’ (red lines), as well as ‘weak conformity’ (orange lines) and ‘anti‐conformity’ (pink lines). Using an error rate (green dashed line) it can also approximate empirical patterns of behaviour [here that of Danchin *et al*. (2018) in the fruit fly] whereby individuals depart from unanimous majority behaviour. In effect, perfect conformity when getting close to 100% adopting the majority behaviour is highly unlikely in actual organisms.

A few decades after Asch's experiments, biologists incorporated conformity into the study of cultural evolution, i.e. the change of culture over time (Claidière & Whiten, 2012; Haun, van Leeuwen & Edelson, 2013; van Leeuwen & Haun, 2013; Whiten, 2019, 2021) where culture is the phenotypic variation that is inherited through social learning (Danchin *et al*., 2018). Integrating socially transmitted information into a population genetics framework raised the question of how individuals use social information to maximize fitness (Cavalli‐Sforza & Feldman, 1981; Boyd & Richerson, 1985). This predicts that such socially aware individuals would be equipped with a wide range of social transmission biases that dictate whom they copy and when (Feldman, Aoki & Kumm, 1996; Henrich & Boyd, 1998; Henrich & McElreath, 2003; Laland, 2004; Enquist, Eriksson & Ghirlanda, 2007; Wakano & Aoki, 2007; Kendal, Giraldeau & Laland, 2009). For instance, individuals could preferentially copy prestigious models (prestige bias), or models that look like themselves (similarity bias), or even – our focus here – models that display the majority behaviour (conformist bias, or *conformity* for short). A rigorous definition of conformity was established early on by Boyd & Richerson (1985): an individual is *conformist* if it is more likely to adopt the majority behaviour than if it copied a demonstrator picked at random (Morgan & Laland, 2012; Muthukrishna, Morgan & Henrich, 2016; see Fig. 1). Given the right conditions, this ‘over‐copy‐the‐majority’ rule could be highly profitable.

Subsequent evolutionary models examined conditions favouring different learning biases. Conformity's ‘sweet spot’ turned out to be a spatially variable environment with migration, since conforming helps migrants to adopt the locally adaptive behaviour quickly (Boyd & Richerson, 1985; Henrich & Boyd, 1998; reviewed in Aoki & Feldman, 2014). More generally, conformists perform better when environmental change is slow, social learning is easy (relative to individual learning), transmitted traits have high adaptive value, and fitness payoffs vary greatly with space (Nakahashi, 2007; Kendal *et al*., 2009).

Conformity adds robustness to the transmission process overall (Dindo, Whiten & de Waal, 2009; Danchin *et al*., 2018; Lachlan, Ratmann & Nowicki, 2018). A conformist learner is unlikely to pick up a one‐off behavioural mistake, since its decision is based on many demonstrators. Yet this redundancy also dampens innovation (Sterelny, 2006), and in rapidly changing environments it may cause a carryover of old behaviours that are no longer adaptive if the individuals are not flexible enough to adapt at the same speed as the environment changes (Aplin, Sheldon & McElreath, 2017). In the context of cultural evolution, this can in turn slow down the response to environmental change and thus promote the maintenance of outdated maladaptive behaviours (Whitehead & Richerson, 2009). In practice however, a mix of conformity and individual learning may allow populations to switch to newly adaptive behaviours. This was shown by Aplin *et al*. (2017) in an experimental study on great tits (*Parus major*): by manipulating behaviour rewards, they documented that both individuals and populations were able to move away from established, now‐suboptimal behavioural traditions. The takeaway message is that conformity can catalyse a behavioural change that is initially driven by individual learning. A related point was made by Perreault, Moya & Boyd (2012), in providing theoretical evidence that conformity is expected to be a universal feature of social learning when individual learning is also in play.

Given the right conditions, conformity can stabilize any trait – adaptive or not (Laland, 1994). For example, in some models conformity can foster cooperation if interactions and learning are spatially constrained in the population, thus giving rise to ‘local clusters’ of cooperators (Peña *et al*., 2009; Mengel, 2009; Molleman, Pen & Weissing, 2013*a*). Instead, in large homogeneous populations, cooperation is diluted, and conformity can block its evolution (Lehmann & Feldman, 2008; Molleman, Quiñones & Weissing, 2013*b*). Similarly, conformity can coevolve with other cultural or genetic traits such as altruism (Lehmann & Feldman, 2008) or the ability to copy (Wakano & Aoki, 2007). Empirical studies to verify these predictions remain needed, for instance to study how conformity interacts with group size to affect traditions of cooperation.

Crucially, conformity can generate a stable combination of within‐group homogeneity and between‐group heterogeneity. Indeed, conformity fixes a given group on a single socially learned tradition that remains stable over generations, making the tradition resistant to erosion and robust to invasion of alternative variants. Importantly, different groups can fix on different traits depending on which trait is locally optimal, cultural drift or some specific initial conditions. This is illustrated by patterns of tool use among chimpanzees (*Pan troglodytes*) from the Taï National Forest (Ivory Coast). These chimpanzees crack *Coula edulis* nuts with stone and/or wooden hammers and use roots as anvils. Which tools they use depends on the community they live in, and is culturally transmitted. Despite high levels of female intergroup migration, differences between neighbouring groups persist over time with incoming females conforming to their new group‐specific tradition (Luncz & Boesch, 2014).

### (1) Looking for normative conformity in non‐human animals

All the evolutionary models mentioned above relate to *informational* benefits of conformity. They depict cases where the majority behaviour is a local fitness‐maximizing social cue. Hence, predicting the evolutionary success or failure of conformity rests on the statistical concept of *Bayesian inference*, as made explicit by Perreault *et al*. (2012). All models thus follow more or less the same reasoning, at least implicitly. They (*i*) take a naïve individual seeking the fitness‐maximizing alternative amongst several behaviours; (*ii*) assume that the best behaviour is selected for so that it increases in frequency; (*iii*) hence, conclude that the most‐displayed behaviour should be the best, provided that the demonstrator population had enough time to undergo selection. Although this reasoning holds under many circumstances, it may fail when the environment is too unstable for the demonstrator population to have reached the best strategy.

In the normative case, benefits instead occur when the population itself rewards a given behaviour (the norm), or punishes non‐compliers. Administering these rewards or punishments is often costly, which impedes the evolution of such social machinery. However, conformity can overcome this evolutionary obstacle so that hardwired conformity allows costly social norms to evolve, implying that conformity might be responsible for the emergence of a reward‐and‐punishment culture (Henrich & Boyd, 2001). Once established, costly social norms can even coevolve with conformity (Gúzman, Rodríguez‐Sickert & Rowthorn, 2007) although such associations are harder to obtain than previously thought (van Cleve, 2016). Since conformity also accounts for the stable combination of intergroup heterogeneity and intragroup homogeneity, it can in turn trigger cultural group selection (Boyd & Richerson, 1985).

Until now, informational benefits have been the dominant explanation for conformist behaviour in evolutionary biology (for an extended discussion see Claidière & Whiten, 2012). Nonetheless, in a non‐conformity context rewards or punishments for complying (or not) with group‐specific rules or norms have been documented in animals (Singh & Boomsma, 2015; reviewed by Raihani, Thornton & Bshary, 2012) particularly in insects and non‐human societies where it seems to maintain intense levels of cooperation, suggesting the existence of normative benefits in non‐human animals. We thus suggest that the normative channel deserves more attention.

### (2) Viability of conformity in strategic settings

Is it always beneficial to copy the majority? As a preliminary answer to this question, consider how you, as a conformist individual, might fare in the following three scenarios.

(1) Choosing between two plentiful food sources, each potentially healthy or poisonous.

(2) Choosing between two identical but limited‐supply food sources.

(3) Choosing between two paths to avoid a predator that preys preferentially on smaller groups.

Scenario 1 is a straightforward win for informationally driven conformity: if a majority of people feed from a given food source, it probably means that it is healthy, so conforming is a good bet. That changes, however, in scenario 2: since the food sources are in limited supply, the more individuals feed from one, the less food remains for you there. Here, conforming will not work well: the situation calls for *not* copying the majority, and you would be better off by taking the less‐popular option. In scenario 3, conformity makes a comeback: the more individuals take a given path, the less likely you are to be attacked by also taking that path (Cresswell & Quinn, 2011). To improve your chances of survival you should learn which is the safe way according to the number of individuals using it, i.e. copy the majority.

The crucial difference between these scenarios is how the behaviour of others affects your fitness. In scenarios 2 and 3 the viability of each option depends on what others do – neither option is intrinsically better independently of the actions of others. Economists call such interactions *strategic*. In scenario 2, those you copy affect your fitness *negatively*. Such behaviours are called *strategic substitutes*, which means that the attractiveness of an option decreases as more individuals choose it (negative frequency dependence). In scenario 3, those you copy affect your fitness *positively* – such behaviours are called *strategic complements*, which means that the attractiveness of an option increases as more individuals choose it (positive frequency dependence) (Bulow, Geanakoplos & Klemperer, 1985). Scenario 1 is non‐strategic as there *is* an intrinsically better option (the healthy one) and the behaviour of others affects your fitness only insofar as it provides social information on what that better option is.

These scenarios sketch a qualitative result: conformity seems to thrive only when behaviours are strategic complements, or non‐strategic. While a rigorous theoretical exploration may be needed to confirm this simple intuition, it suggests that strategic complements or substitutes may provide a useful lens to understand why conformity evolves for some traits but not others. Further research is needed, both on theoretical and empirical fronts, to investigate this potential link between conformity and strategic settings.

### (3) Information acquisition, the value of observations, and memory

As a matter of fact, the classical formalisms used to model information acquisition largely are idealized. Models commonly assume a simple group structure where individuals observe *simultaneously everyone else* in their group, and then infer the majority behaviour from this far‐reaching, sweeping look. More realistically, observations in fact are gathered *sequentially* from a *subset* of the whole population (Morgan, Acerbi & van Leeuwen, 2019), as in a recent study on chimpanzees (Watson *et al*., 2018). Organisms must deduce the majority behaviour from such imperfect samples, probably by using individual learning and memory, which none of the models of conformity to date has implemented. Different environments could promote different ways to deal with information. In unstable environments for instance, older observations may be discounted in favour of recent ones, since reliance on past observations could cause deleterious behavioural inertia. Regarding the size of the sample of demonstrators, Denton *et al*. (2020) showed that evolutionary dynamics can be more complex than previously thought when there are more than three role models, as in Boyd & Richerson's (1985) classical treatment.

Similar arguments hold for how to integrate space, prestige or other characteristics into the evaluation of observation. Should an immigrant completely ignore information from its home environment in favour of local practices? Should it give more weight to demonstrators like itself? Is a prestigious demonstrator worth two, 10, or a 1000 ordinary ones? Mesoudi (2018) addressed similar questions by modelling immigrant conformist acculturation. Basically, this comes down to studying the interaction between conformity and other biases, such as prestige, success, locality, ethnicity, age, sex, etc. The core problem is to determine the value of adding a single demonstrator into the decision process, based on that demonstrator's personal characteristics relative to the observer. This is both a theoretical and an empirical question that needs to be explored.

Finally, while most current studies concern binary choices, natural situations usually involve many more options. Expanding the binary approaches would raise empirical challenges (what would occur when the majority is still an absolute minority, as in a 40–30–30 split?) and theoretical ones. A first unanswered problem is to devise a suitable learning rule (an S‐shaped probability, symmetric in all behaviours) when there are more than two behaviours to choose from, or even when traits are continuous. These issues remain under‐explored for the moment, with a few exceptions (e.g. Nakahashi, Wakano & Henrich, 2012; Mesoudi, 2018). In the next section we outline a possible step in this direction.

### (4) Conformity with more than two choices: a functional form and a statistical test

The canonical mathematical model for conformist transmission is the sigmoid curve (Fig. 1A, red lines) proposed by Boyd & Richerson (1985) in their initial work on conformist transmission and cultural evolution (Fig. 1A). Their model considers the case with two traits, where learners are exposed to three demonstrators and exhibit a positive frequency‐dependent bias. If we call *q* the proportion of trait A in the population, and 1 − *q* the proportion of trait B, then Boyd & Richerson (1985) defined the probability *p* that an individual will adopt trait A as

(1) $$p\left(q\right)=q+\mathrm{Dq}\left(1-q\right)\left(2q-1\right)$$

where *D* is a parameter between 0 and 1 that expresses the strength of the positive frequency‐dependent bias. This model conveniently encapsulates the essential feature of the conformist bias, i.e. that an individual is disproportionately likely to adopt the majority variant. However, as can been seen from Fig. 1A, it covers only a small fraction of all possible S‐shaped curves. In particular, it cannot account for very steep slopes around *p* = 0.5. This is because Boyd & Richerson's learners only observe a small sample of demonstrators, preventing them from accurately identifying the majority behaviour. Yet, empirical work has shown that individuals can be surprisingly efficient in detecting majorities around 50%, leading to a sharp behavioural response to demonstrator frequencies that can look more like a step function than a mild sigmoid curve (Aplin *et al*., 2017; Danchin *et al*., 2018). Boyd & Richerson's (1985) framework could in theory be extended to account for this, but it quickly becomes analytically untractable when the number of demonstrators or traits becomes large. Therefore, does a simple model exist which can accommodate these very steep slopes, and (ideally) that could also be generalizable to the case with any number of traits?

One such model could be derived from McFadden (1973)’s discrete choice model, which is commonly used in economics to describe consumer choices. The probability of adopting trait A takes the logistic form

(2) $$p\left(q\right)=\frac{q^{\beta }}{q^{\beta }+{\left(1-q\right)}^{\beta }},$$

where *β* is a real‐value parameter expressing the strength of the conformist bias. This model can be micro‐founded by considering that learners pick up the most common trait among a large pool of observed demonstrators but may be subject to random errors. [Specifically, *p*(*q*) takes this form when the learner chooses the trait *j* which maximizes ln(*q*_*j*_) + *ε*_*j*_ where the *ε*_*j*_ are independent and identically distributed Extreme Value Type‐I, with *β* a scale parameter.] In this case, the smoothness of the learning curve is a result of these behavioural errors, as opposed to the Boyd & Richerson (1985) model where it comes from small sampling errors. As can be seen from Fig. 1B, this alternative model is very comprehensive for describing milder to stronger forms of conformity in Boyd & Richerson's sense. One can also show numerically that these curves resemble those that can be obtained by extending Boyd & Richerson's (1985) model to an arbitrary number of demonstrators. Furthermore, it also accommodates other learning rules, which sometimes have been called ‘weak conformity’ and ‘anti‐conformity’ (see Whiten, 2019). Specifically,

*β* > 1 corresponds to conformist transmission in Boyd & Richerson's (1985) sense;

0 < *β* < 1 corresponds to weak conformity;

*β* < 0 corresponds to anti‐conformity.

These three cases also suggest a simple statistical test for conformity. Assuming that one has data on individual behavioural responses as a function of demonstrators' behavioural frequencies *q*, testing for whether these behavioural data exhibit conformity is then straightforward: the data must fit the model for a value of *β* greater than 1. To test this, simply note that

(3) $$\ln\left(\frac{p\left(q\right)}{1–p\left(q\right)}\right)=\beta \;\ln\left(\frac{q}{1–q}\right),$$

i.e. that there is a linear relationship between the log odds of the response probabilities and the log odds of the demonstrators' frequencies, with the associated slope being *β*. The value of *β* can thus be obtained by performing a simple linear regression, since the log odds are immediately available from the data. Whether this coefficient is significantly above 1 should be evidence for conformist bias.

A convenient feature of this model is that it can be easily extended to any number of traits,

(4) $$p_{i}\left(q\right)=\frac{q_{i}^{\beta }}{\sum_{j}q_{j}^{\beta }},$$

where *q* is now a vector of population shares *q*_*j*_ for each trait *j*. A corresponding statistical test can then be derived from the following equalition, valid for any two traits A and B:

(5) $$\ln\left(\frac{p_{A}\left(q\right)}{p_{B}\left(q\right)}\right)=\beta \;\ln\left(\frac{q_{A}}{q_{B}}\right).$$

Lastly, both Boyd & Richerson's (1985) mathematical model and the one we propose above fail to account for individual departures from unanimous majorities (i.e. a few learners picking up trait B when 100% of demonstrators show trait A; see Fig. 1A). Yet, such occurrences were documented as early as Asch (1955) and have been corroborated in many animal studies of conformity (e.g. Pike & Laland, 2010; Battesti *et al*., 2015; Aplin *et al*., 2017; Danchin *et al*., 2018). This phenomenon could for instance be evidence of major cognitive limitations, or of important heterogeneities within populations regarding learning rules. In any case, this feature seems ubiquitous and may lead to false negatives when using the statistical test suggested above. However, a simple extension of the model above can address it. Consider that the learner chooses a trait at random with probability *α*, and chooses a trait using the conformist rule *p*(*q*) above with probability 1 – *α*. In the case with two traits, the probability of adopting trait A becomes

(6) $$p^{\prime }\left(q\right)=\frac{\alpha }{2}+\left(1–\alpha \right)\frac{q^{\beta }}{q^{\beta }+{\left(1–q\right)}^{\beta }}\;.\;$$

Once again, one can estimate this equation on behavioural data and check whether *β* > 1 for evidence of conformist transmission in Boyd & Richerson's (1985) sense.

## IV. CONFORMITY IN NON‐HUMAN ANIMALS

### (1) Behavioural ecology

Boyd & Richerson's (1985) conformist transmission model has recently attracted a lot of attention in behavioural ecology (e.g. Brown & Laland, 2002; Pike & Laland, 2010; Nelson & Poesel, 2014; Aplin *et al*., 2015*b*, 2017; Battesti *et al*., 2015; Danchin *et al*., 2018; Lachlan *et al*., 2018; Ayoub, Armstrong & Miller, 2019), the domain of evolutionary sciences that studies the evolution of behaviour (Davies & Krebs, 1984; Danchin, Giraldeau & Cézilly, 2008). However, although conformity in humans rapidly attracted attention, it remained largely overlooked in non‐human animals for a long time, with the first studies of animal conformity being published in the early 2000s, more than 50 years after Asch's studies. Although non‐human animal conformity is now becoming a hot topic, the junction with the domain of human conformity remains to be made to allow the full study of the evolutionary origins of human conformity.

In the non‐human animal literature, conformity is often defined as ‘behaving like, or copying, the majority’ which is in many cases a simplification of Boyd & Richerson's (1985) ‘disproportionate likelihood of adopting the majority strategy’ definition. At the very least, this broader definition raises several important issues that are also subject to recent debate. First, the definition ‘copy the majority’ does not specify whether the majority concerns the larger number of individuals performing a behaviour or whether it concerns the behaviour that is most frequently displayed (van Leeuwen *et al*., 2015). Indeed, it is interesting to notice that this is not specified in Boyd & Richerson's (1985) model either. From an evolutionary standpoint, the majority should refer to the number of individuals performing a behaviour. This allows an outsider to grasp easily what is the appropriate behaviour under given circumstances. Otherwise, there might be only a few individuals over‐displaying a given behaviour, making it the most frequent, but not the one adopted by the majority of individuals in the population (van Leeuwen *et al*., 2015; Acerbi *et al*., 2016). However, in some cases, it can also be beneficial if the behaviour with the highest frequency is adopted; namely when particularly highly skilled or successful individuals perform one behaviour especially frequently. For example, when great tits trained to open a puzzle box to get a food reward were released into the wild, these few skilled individuals frequently opened the slider of the puzzle box in a specific way and this technique was adopted by other individuals in the population. Thus, conforming with respect to the frequency of options performed may be as adaptive as conforming with respect to the majority of individuals (Aplin *et al*., 2015*a*). Similarly, models showed that both the number of individuals and the frequency of the behaviour can lead to the same sigmoidal conformity curve (Smaldino, Aplin & Farine, 2018). It is likely that if at the beginning the most frequent behaviour is copied, soon a majority of individuals in the population will also exhibit this behaviour. Second, this simplified definition of ‘copy the majority’ is sometimes used only for cases where an individual performing behaviour A *changes* to behaviour B to follow the majority (e.g. Cialdini & Goldstein, 2004; Cherng *et al*., 2014; Haun, Rekers & Tomasello, 2014). For example, wild male vervet monkeys (*Chlorocebus pygerythrus*) that migrate to a new group will abandon personal foraging preferences in favour of the new group norms (van de Waal, Borgeaud & Whiten, 2013). This restriction that behaviour A must first be learned before switching to B seems unnecessarily restrictive as even naïve individuals can show conformist behaviour after observing others. Last but not least, it is important to focus on the fact that frequency‐dependent copying *without* disproportionately likely copying of the most common variant cannot foster stable local traditions in which all group members adopt the same behaviour. This is illustrated in Fig. 1A where Boyd & Richerson's (1985) definition of conformity corresponds to the grey areas, while the common broader definition of conformity would also consider strategies in the white areas as conformist, despite the fact that such strategies would invariably rapidly drive populations towards a stable equilibrium with no majority (i.e. at 0.5). Thus, the ‘disproportionate likelihood of adopting the majority strategy’ is crucial to study and compare conformity both in non‐human animals and humans.

Although research on non‐human animal conformity is relatively recent, there is some evidence for conformity in several taxa from insects to great apes and in various contexts including foraging, song learning, problem‐solving tasks, tool use and mate choice (Table 1). Unfortunately, these studies use very different kinds of definition of conformity ranging from a ‘copy the majority’ rule, over Aschian conformity where a personal preference/behaviour is replaced by the majority preference/behaviour to Boyd & Richerson's (1985) definition of conformist transmission. Thus, the field would benefit from clearer definition and consistent use of terminology to allow for comparisons across species and studies. More generally, Table 1 does not include the well‐studied phenomena of herding or shoaling and other processes of collective behaviour [for instance, coloniality in birds (e.g. Siegel‐Causey & Kharitonov, 1990; Buckley, 1997; Danchin & Wagner, 1997; Danchin, Boulinier & Massot, 1998; Dukas & Edelstein‐Keshet, 1998; Rolland, Danchin & de Fraipont, 1998; Doligez *et al*., 1999, 2003; Barta & Giraldeau, 2001; Brown & Bomberger Brown, 2001; Serrano *et al*., 2001; Boulinier *et al*., 2002; Doligez, Danchin & Clobert, 2002; Eberhard, 2002; Parejo *et al*., 2007; Varela, Danchin & Wagner, 2007)] that are not usually called conformity, despite the fact that such processes can be envisioned as forms of conformity in space as long as they involve social learning, which was clearly suggested by several authors from both observational and experimental data (Danchin *et al*., 1998; Boulinier *et al*., 2002; Doligez *et al*., 2002, 2003; Parejo *et al*., 2007).

**Table 1:** Reported examples of conformity in the animal kingdom. We include only instances when the authors explicitly referred to conformity (see Section IV.1 for comments on this choice).

Context	Species	Working definition used	References
Cooperation	Ant (*Paratrechina longicornis*)	“Conformist group members align their actions with those of their neighbours” (p. 2)	Gelblum *et al*. (2015)
Oviposition site	Fruit fly (*Drosophila melanogaster*)	“The tendency to disproportionately adopt the most commonly encountered social information” (p. 84)	Battesti *et al*. (2015)
Mate choice	Fruit fly (*Drosophila melanogaster*)	“An exaggerated tendency to copy the majority” (p. 362)	Danchin *et al*. (2018)
Shoaling	Mosquitofish (*Gambusia holbrooki*)	No clear definition	Herbert‐Read *et al*. (2013)
	Guppy (*Poecilia reticulata*)	“Positive frequency‐dependent social learning” (p. 917)	Day *et al*. (2001);
		“Many animals are disproportionately likely to adopt via social learning the behaviour of the majority” (p. 41)	Brown & Laland (2002);
		“Strong compulsion for individuals within social groups to remain in close contact and look and behave similarly” (p. 95)	Brown & Irving (2014)
	Rummy‐nose tetra (*Hemigrammus rhodostomus*)	“Tendency to follow the majority of their neighbours nonlinearly” (p. 1)	Lecheval *et al*. (2018)
Personality	Solitary crab (*Carcinus maenas*)	“Animals compromise their own behaviour to the level of a certain behaviour displayed by another individual or a group” (p. 131)	Fürstbauer & Fry (2018)
	Eurasian perch (*Perca fluviatilis*)	“Behaving uniformly” (p. 501)	Hellström *et al*. (2011);
		No clear definition	Magnhagen (2012)
	Gouldian finch (*Erythrura gouldiae*)	“Individuals will tend to synchronize their behaviour in time and space, altering their behaviour in line with their group mates, and potentially suffering consensus costs” (p. 26)	King *et al*. (2015)
	Marmoset (*Callithrix jacchus*)	No clear definition	Koski & Burkart (2015)
Song	White‐crowned sparrow (*Zonotrichia leucophrys*)	“When a young pupil models its song(s) on those of one or more tutors” (p. 433)	Nelson & Poesel (2009);
		“Disproportionate tendency to copy the most common behavioural variant” (p. 1742)	Nelson & Poesel (2014)
	Swamp sparrow (*Melospiza georgiana*)	“Disproportionate tendency to copy the majority” (p. 2)	Lachlan *et al*. (2018)
	Indo‐Pacific bottlenose dolphin (*Tursiops aduncus*)	“Being increasingly likely to adopt the most frequent behaviour” (p. 6)	Cantor & Whitehead (2013)
	Humpback whale (*Megaptera novaeangliae*)	“Being increasingly likely to adopt the most frequent behaviour” (p. 6)	Cantor & Whitehead (2013)
	Sperm whale (*Physeter macrocephalus*)	“Learn preferentially the most common codas” (p. 1)	Cantor *et al*. (2015)
Tool use	Japanese macaque (*Macaca fuscata*)	“Immature individuals should adopt the same type of stone‐directed activities as most of the older group members” (p. 124)	Leca *et al*. (2010)
	Chimpanzee (*Pan troglodytes*)	“A powerful tendency to discount personal experience in favour of adopting perceived community norms” (p. 738)	Whiten *et al*. (2005);
		No clear definition	Whiten *et al*. (2007);
		“Personal knowledge was dropped in order to adopt the behaviour of the group” (p. 650)	Luncz & Boesch (2014)
Foraging	Nine‐spined stickleback (*Pungitius pungitius*)	“Positive frequency dependent social learning where the probability of acquiring a trait increases disproportionately with the proportion of other individuals performing it” (p. 466)	Pike & Laland (2010)
	Threespined stickleback (*Gasterosteus aculeatus*)	No clear definition	Webster & Hart (2006);
		No clear definition	McDonald *et al*. (2016)
	Zebrafish (*Danio rerio*)	“Copy the majority strategy” (p. 1519)	Zala *et al*. (2012);
		“Individuals will appear to disproportionately copy the most common behavioural choice demonstrated by their group” (p. 164)	Ayoub *et al*. (2019)
	Great tit (*Parus major*)	“Tendency for naïve individuals to disproportionately adopt the most common behaviour (‘conformist transmission’) and by a tendency for individuals with experience of both techniques to change their behaviour to match the common variant (‘conformity’)” (p. e5)	Aplin *et al*. (2015*a*);
		“Disproportionate tendency to copy the most common behavioural variant” (p. 7830)	Aplin *et al*. (2017)
	Norway rat (*Rattus norvegicus*)	“Changing one's behaviour to match that of others” (p. 769)	Jolles *et al*. (2011)
	Indo‐Pacific bottlenose dolphin (*Tursiops aduncus*)	“Being increasingly likely to adopt the most frequent behaviour” (p. 6)	Cantor & Whitehead (2013)
	Humpback whale (*Megaptera novaeangliae*)	“Being increasingly likely to adopt the most frequent behaviour” (p. 6)	Cantor & Whitehead (2013)
	Redfronted lemur (*Eulemur rufifrons*)	“Adoption of the group's norm, despite being in principle able to behave differently, or overriding of individually learned by socially acquired information” (p. 506)	Schnoell & Fichtel (2012)
	Capuchin monkey (*Cebus apella*)	“Conform to the foraging preferences of their closest social partners, despite having the knowledge of alternative techniques” (p. 4)	Dindo *et al*. (2009);
		No clear definition	Crast *et al*. (2010);
		No clear definition	Franz & Matthews (2010)
	White‐faced capuchin monkey (*Cebus capucinus*)	“The tendency for individuals to preferentially exhibit behavioural alternatives that they witness most frequently in their peers, or to exhibit the behaviours that are performed by peers who are considered most prestigious or successful, or those peers with whom they have the highest quality social relationships” (p. 706)	Perry (2009)
	Vervet monkey (*Chlorocebus pygerythrus*)	“Conformity to local behavioural norms” (p. 483)	van de Waal *et al*. (2013)
	Chimpanzee (*Pan troglodytes*)	“Follow‐the‐majority (= the number of animals in a group performing a specific behaviour increases, so does the likelihood of a naïve individual adopting that same behaviour, thus driving the preservation)” (p. 1195)	Hopper *et al*. (2011);
		“The increased likelihood for learners to end up not with the most frequent behavior but rather with the behavior demonstrated by most individuals” (p. 727)	Haun *et al*. (2012);
		“Foregoing a pre‐existing behaviour in favour of adopting one demonstrated by a majority of conspecifics” (p. 407)	Watson *et al*. (2018)

All these considerations suggest that there is probably more evidence for the existence of informational conformity in animals than usually thought. This vast breadth of taxonomies and contexts (Table 1) raises the question of the evolutionary origin of conformity. In itself, this breadth suggests convergent selection for conformity rather than a homologous capability (Laland, Atton & Webster, 2011). However, we are still lacking a general framework by which conformity may have emerged in the first place during the course of evolution.

### (2) Conformity in mate choice and the Fisher runaway process

Our review of the literature on non‐human animal conformity has revealed a quasi‐absence of evidence for conformity in the context of mate choice (Table 1, with the nuance we introduce in Section V). The only clear example of conformity in mate choice in any animal including humans is a study in fruit flies (*Drosophila melanogaster*) where females develop mating preferences for a certain male phenotype in a conformist manner (Danchin *et al*., 2018). In that study, as long as there was a majority of demonstrator females copulating with males of a given phenotype, observer females copied this choice and developed a similarly significant bias for males of that specific phenotype whatever the level of majority in the population. As a result, the response function of observer females followed a step function (as the green dotted curve of Fig. 1B), with females learning equally well to prefer the most commonly chosen male colour whatever the level of majority, which in that study varied experimentally from 100% to only 60%. However, a certain number of individuals did not copy the majority for whatever reason (e.g. being anti‐conformists or individual learners). A simple model also suggested that this strong conformity in mate choice might generate surprisingly long‐lasting traditions of preferring one male phenotype at the local scale (Danchin *et al*., 2018).

The *Drosophila* result on conformity calls for an evolutionary explanation. Interestingly, conformity in the context of mate choice is reminiscent of the well‐known Fisher runaway process (Fisher, 1930). According to this process, both male traits and female preferences are supposed to be under genetic control. Under such conditions, as soon as females develop some preference, females tend to mate with their preferred male phenotype, which automatically generates a correlation between the male trait and the female preference. This generates a linkage disequilibrium of purely statistical nature (Bailey & Moore, 2012). This statistical linkage participates in what we now call ‘inclusive heritability’, i.e. parent–offspring resemblance due to genetic or non‐genetic information being transferred from parents to offspring (Danchin & Wagner, 2010; Danchin *et al*., 2011). Such resemblance can involve DNA sequence variation (which is probably partly valid for the male trait) but could also result from the cultural transmission of female preference. What matters is that females transmit their preference to their daughters, and fathers their trait to their sons, so that the information responsible for the two traits (e.g. male trait A, and the preference for A) are transmitted jointly. Hence, the female trait becomes statistically associated with the male trait, producing a self‐reinforcing process called the Fisher runaway process (Fisher, 1930). Based on this understanding of the Fisherian process, we now propose and provide a generic model for a possible two‐step evolutionary scenario rooted in this process, which unfolds at two different spatial scales.

**Fig. 2:**
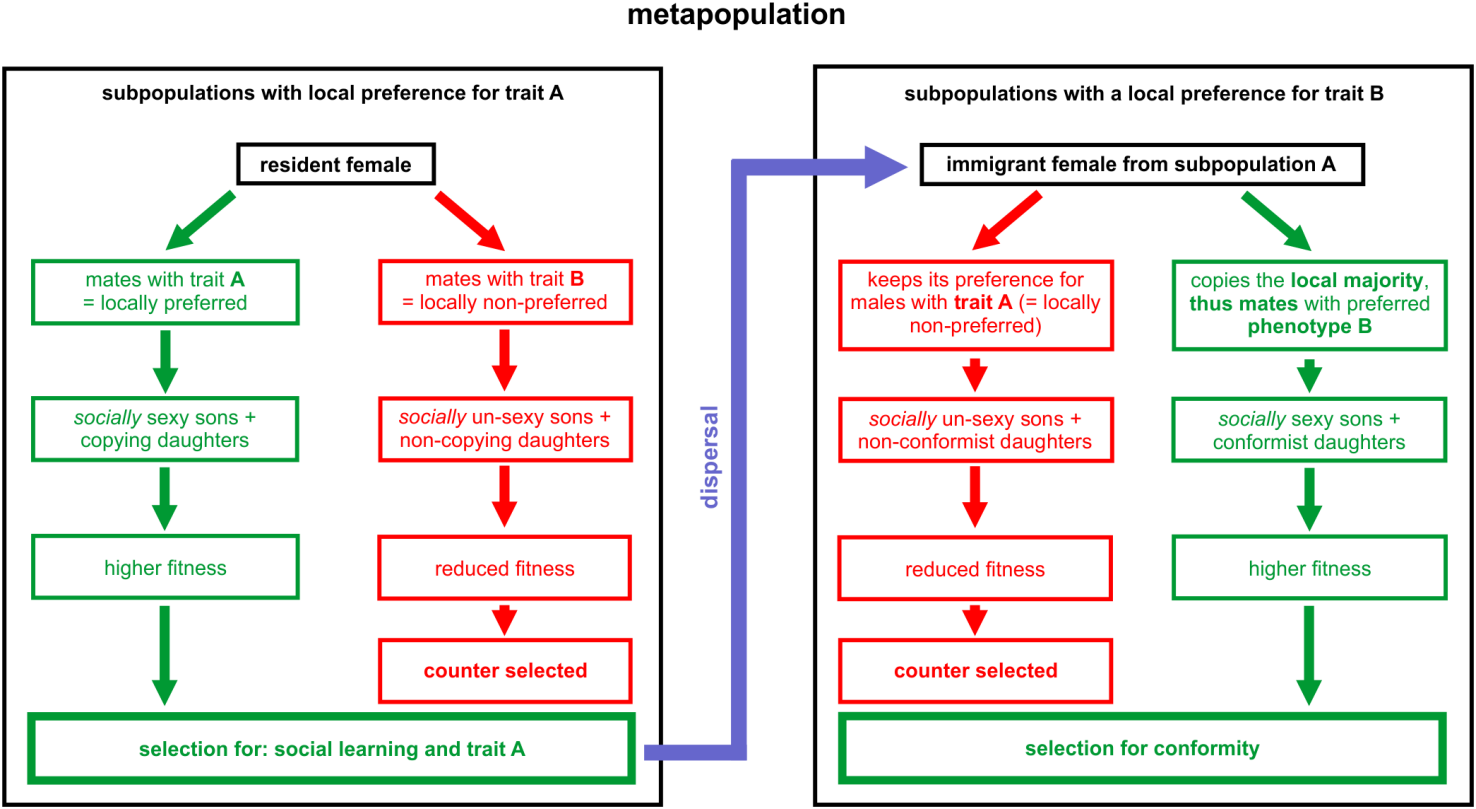
Social learning and conformity in mate choice are produced by the Fisher runaway process within metapopulations. As females are usually the choosy sex, we illustrate that option. In some subpopulations, the majority of females mate with males of trait A while in others they prefer males of trait B. Costly options (mating with the locally non‐preferred male phenotype) are shown in red and beneficial options (mating with the locally preferred male phenotype) are in green. Within subpopulations this selects for one male phenotype and social learning (left panel), but at the scale of a metapopulation dispersal leads to selection for ‘copy the majority’, that is for conformity (right panel).

Let us first imagine an ancestral metapopulation with two heritable male phenotypes A and B with equal fitness (as is the case, for instance, in Danchin *et al*., 2018). There is no initial female preference for one of these male phenotypes, but some inclusively heritable variation in females' tendency to copy others (due to the inheritance of genetic or non‐genetic information). In a given subpopulation, chance may sometimes lead to more females mating with, for instance, A males. As soon as such a majority becomes detectable, copier females tend to mate more often with A males. As a result, they will tend to have A sons and daughters that tend to copy the choices of their elders, implying that they reject B males (left panel, Fig. 2). At the scale of the subpopulation, because of the Fisher runaway process, this reinforces both the proportion of A males and the tendency socially to learn to prefer A males. On the other hand, females of the initial subpopulation choosing B males (i.e. mostly non‐copiers) would have B sons and mostly non‐copier daughters that mate randomly with A and B males. So, at the beginning, while copier females tend to amplify the proportion of A males and the tendency to copy, non‐copier females produce sons in proportion to the frequency of each male type in the subpopulation, while producing non‐copier daughters. Thus, non‐copier females do not affect the proportion of male types.

Thus, at the scale of the subpopulation, locally both the proportion of A males and of copier females slowly increase at a rate that accelerates with the proportions of copier females in a kind of snowball effect. As a result, non‐copier females mating with B males will have B sons that will be more and more socially unsexy over the course of generations. On the other hand, copier females mating with A males are more and more favoured (Fig. 2, left panel). In summary, at the local scale, this process transitorily selects for A males, and for social learning in mate choice. This process, however, is transitory because social learning becomes neutral after the local disappearance of the B male phenotype (see simulations in Section VI). Note that even if the two male phenotypes initially did not differ in terms of fitness, the trait soon quits neutrality because of the social transmission of mating preferences in females. This is the runaway process.

Now, this logic unfolding within a single subpopulation should simply transitorily select for females preferring A males. Nonetheless, that transitory phase probably generated a correlation between male trait and female preference for it. As this correlation is likely to persist for some time after the disappearance of B males, this implies that if dispersing B males enter that local population the Fisher runaway process will immediately resume. In fact, at the scale of a metapopulation, which consists of subpopulations connected by dispersing individuals, in some subpopulations selection would favour females mating with A by chance (Fig. 2, left panel), and in other subpopulations females mating with B males (Fig. 2, right panel). In such a system, dispersal hampers the local evolution of a heritable preference for A, and favours conformity, as immigrants detecting the local preference and conforming to it have higher fitness (Fig. 2, right panel and Section VI). Hence, it is the spatial structure of the metapopulation with individuals dispersing among subpopulations that generates selection for the more integrated rule of ‘mate with males of the *locally* preferred phenotype’, i.e. for conformity.

Note that this process is superficially similar to a process by which conformity could arise for foraging preferences, but it is different at a deeper level as in the latter case conformity evolves due to natural and not sexual selection as proposed above. Suppose that in the first subpopulation fruit C is nutritious and fruit D is poisonous, while in the second subpopulation there is fruit E, resembling C, that is poisonous and fruit F, resembling D, that is nutritious. A preference for C and a preference for copying the majority would be equally adaptive in the first subpopulation, just as a preference for F and a preference for copying the majority would be equally adaptive in the second subpopulation. However, for dispersers between the two, a preference for copying the majority would continue to be adaptive, while a preference for C, translated to the second environment, might be fatal, as would a preference for F, translated to the first environment. So, conformity would be more adaptive on average than either direct nutritious preference.

Unlike the Fisher process, though, this process would not be self‐reinforcing – conformity would not become more adaptive as it became more common in the population. In addition, its adaptive value would depend on the absence of consistency in the nutritious value of foraging preferences across environments. This may be true in some ecologies but lacks the general autocatalytic runaway character of the Fisher process at the metapopulation scale, which is independent of other characteristics of the environment.

In view of the generality of the Fisher runaway process in sexually reproducing organisms, and in view of its autocatalytic properties, we should expect conformity in mate choice to be far more common than usually understood. In other words, it should have evolved very early in evolution, soon after the moment when sexually reproducing organisms developed enough cognitive capacities to detect the local majority. This raises the question of the links between mate copying and conformity.

Mate copying is a special case of social learning in the context of mate choice in which females build their own preference from observations of another female choosing between male phenotypes. Mate copying can be performed in a conformist manner (Danchin *et al*., 2018), i.e. over‐copying what is preferred by the majority on one hand, and on the other hand conformity in mate choice can be the result of positive frequency‐based mate copying. Unfortunately, very few studies of mate copying have tested whether it is performed also in a conformist fashion, precluding us from studying the links between these two processes. In fact, most studies on mate copying use only a single demonstration for practical reasons (reviewed in Varela, Matos & Schlupp, 2018), thus preventing us from talking about the majority. We suggest that mate‐copying experiments may in fact constitute a specific test of conformity in which the experimental design limits itself to a sample size of demonstrators of 1. As animals have been selected to conform in mate choice (as we developed above), as soon as they see one demonstration, they may interpret it as a majority preference in the population, because chance makes it more likely that this single observation represents the local majority. But the smaller the majority the higher the risk of opting for the locally non‐preferred male phenotype (which corresponds to the black random copying line in Fig. 1B). In nature, however, by observing multiple matings, observer females can considerably increase the quality of their assessment of the local majority and thus can considerably increase their chance of building a preference for the majority male phenotype.

This reasoning would suggest that the rather large literature on mate‐copying experiments may reveal the existence of conformity in mate choice in the many concerned species. Hence, we can predict that a quick way to increase the evidence for conformity in non‐human animals would be to test for conformity in species known to preform mate copying by simply adapting the experimental design in order to be able to show multiple demonstrations in order to manipulate the majority as in the testing of Danchin *et al*. (2018)'s criterion 5. If, as we predict, many such studies provide evidence for conformity in sex, this would support the idea that mate copying and conformity are tightly linked, and that mate copying and conformity in mate choice are two faces of the same coin that jointly accelerate and maintain the evolution of local traditions for preferring a specific male phenotype over generations. Thus, future studies should systematically test for conformity in mate copying. It is thus of prominent importance to design experiments that manipulate the level of majority in order to study the response function of conformity in mate choice in a wide range of species.

## V. FROM FISHER TO HUMAN CULTURE

Beyond the fact that the existence of conformity in numerous animal taxa generates a continuum from non‐human animals to humans, it is necessary to integrate some of the major consequences of the evolutionary scenario presented in the previous section in terms of its potential to foster cultural transmission in general and in humans in particular.

**Fig. 3:**
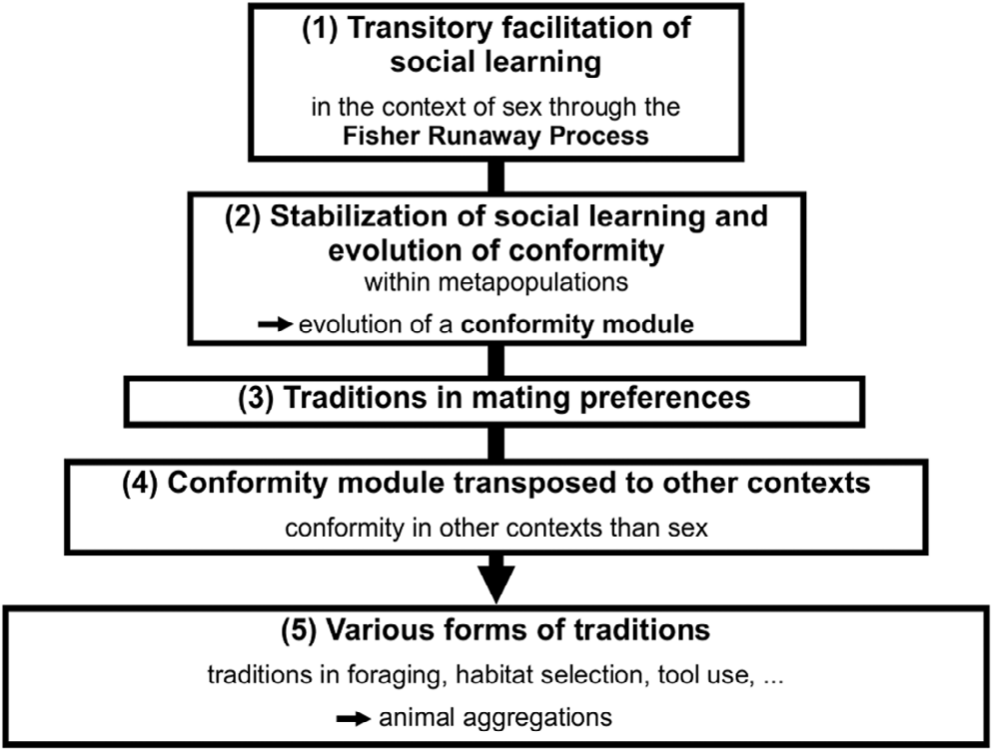
A tentative evolutionary pathway towards culture. From the Fisher runaway process in the context of mate choice to social learning, conformity, traditions in sexual preferences, various forms of traditions including aggregated behaviour, and culture.

As discussed above, the first step in this evolutionary pathway unfolds locally and temporarily favours social learning (Step 1 of Fig. 3). Similarly, the second component results from individuals dispersing among subpopulations within the metapopulation, which stabilizes social learning and leads to the evolution of conformity (Step 2 of Fig. 3). Thus, in highly mobile species, conformist mate choice might have fostered the development of a kind of ‘conformist module’ for detecting the majority and following it in order to mate preferentially with the locally preferred male phenotype. These two first steps are detailed in the previous section and should foster the emergence of local traditions in mating preferences (Step 3 of Fig. 3). In view of the generality of the Fisher runaway process and of the scenario proposed in Section IV.2, these capacities probably evolved as soon as sexually reproducing ancestral species acquired the capacity to detect the majority, so that they should be present in a vast array of species.

However, the story does not end there. We have detailed above that there is some theoretical and empirical evidence that conformity is potent in fostering a cultural process (Fisher, 1930; Sterelny, 2006; Efferson *et al*., 2008; Laland *et al*., 2011; Danchin *et al*., 2018; Lachlan *et al*., 2018). Like norms or punishment, conformity can play the role of a ‘repair mechanism’ to maintain an existing preference/behaviour. Thus, once acquired and potent, the conformity module may have percolated to other contexts such as foraging, problem solving, tool use, etc. (Step 4 of Fig. 3). Alternatively, cognitive processes involved in copying foraging behaviour may have been subsequently co‐opted for use in mate choice. Some mechanisms, however, do not seem easily co‐opted from foraging to mate choice – for instance, the pheromone trails that yield conformity in ant foraging (Sumpter & Beekman, 2003). Another alternative is that conformity emerged independently in foraging and mate choice within lineages. Further studies are needed to rule out the different possibilities. Nonetheless, as in this scenario initial fitness benefits accrue from reproduction, it predicts that social learning should remain efficient during the whole reproductive period, which appears consistent with some non‐human animal and human studies.

The evolution of a conformity module that expanded to other contexts then may have fostered the emergence of local traditions in many domains of behaviour (Step 5 of Fig. 3). This pathway would thus imply that all the reported cultural processes, whatever the domain involved, would find their origin in mate choice and sexual reproduction in the first ancestors that acquired the capacity to detect and comply with the majority, which must have produced the initial trigger of the Fisher runaway process.

## VI. A GENERIC MODEL OF THE EVOLUTION OF CONFORMITY

**Fig. 4:**
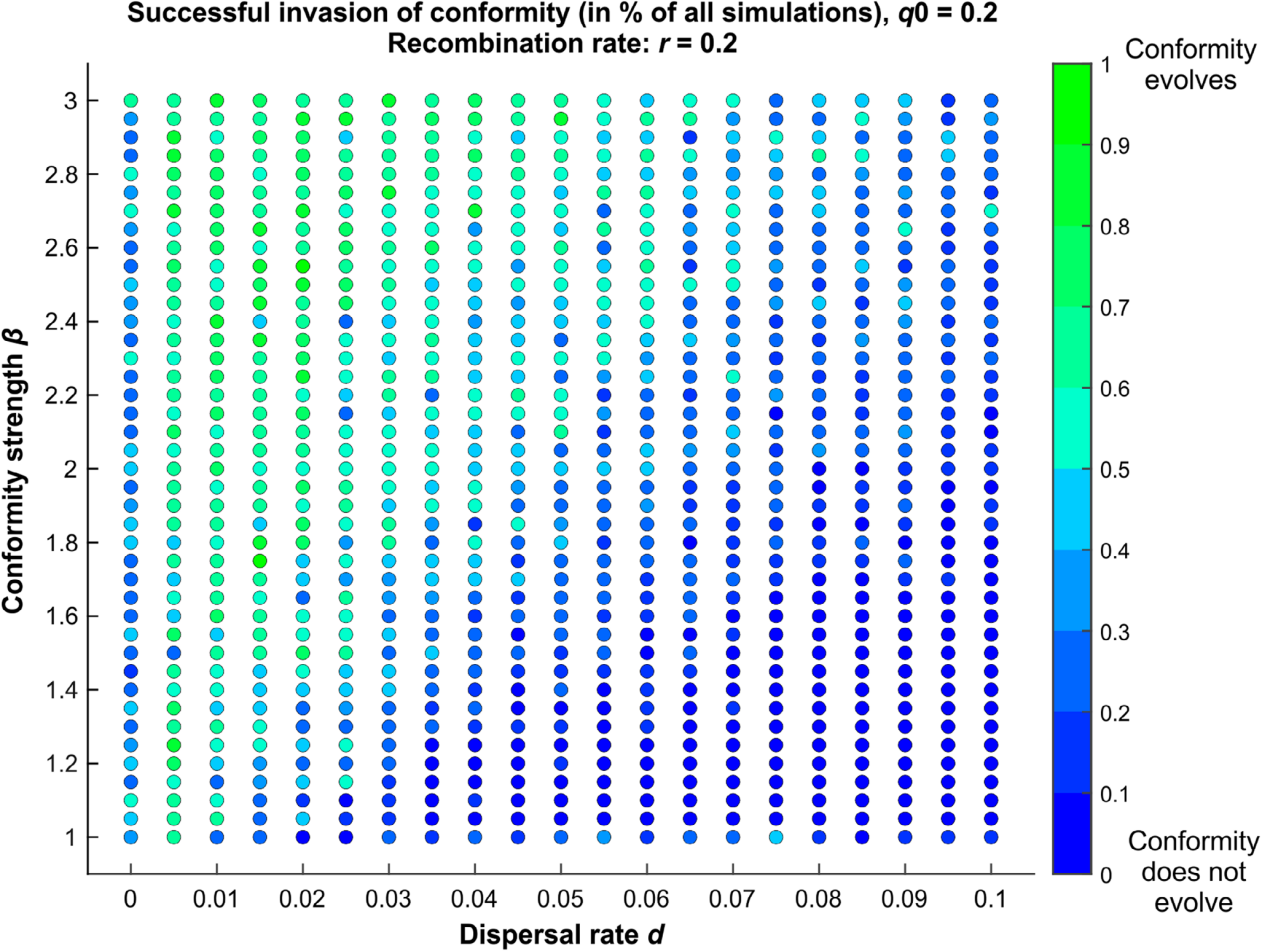
Evolution of conformity in spatially structured metapopulations for different parameter values. Each point is obtained using 20 simulation runs of the evolution of conformity in a population of non‐copiers. Males express either trait A or B. Females are conformist or pick a male randomly. The initial fraction of conformist females is *q*0 = 0.20. The recombination rate is fixed at *r* = 0.20. Conformity evolves when the dispersal rate *d* is not too high compared to the strength of conformity *β*. The code for this model is available at https://github.com/antoine‐jacquet/project‐conformity

**Fig. 5:**
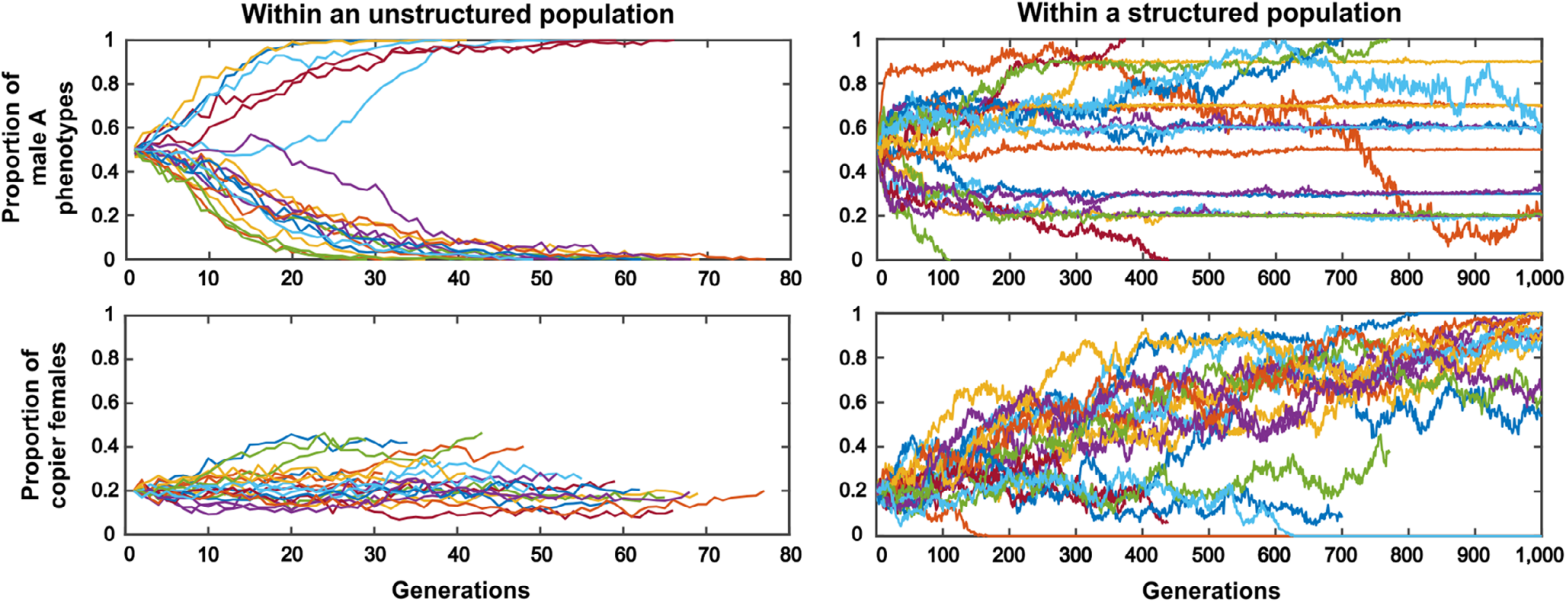
Conformity evolves in spatially structured metapopulations. In a simple simulation model, we found that dispersal among subpopulations within a metapopulation can favour the evolution of social learning and conformity, which are well known to be essential traits for the evolution of long‐lasting local traditions, the main marker of a cultural process. Graphs obtained in simulations of the evolution of conformity in a population of non‐copiers for the set of parameter values: *β* = 2.8, *d* = 0.02, *q*0 = 0.20, *r* = 0.20 (see Fig. 4 for definitions of these variables). Males express either trait A or B. Females are conformist or pick a male randomly. Lower graphs show dynamics of the conformity allele. Upper graphs show male trait dynamics. Left‐hand graphs depict the dynamics within unstructured populations. Male diversity is rapidly lost, and conformity remains neutral. Right‐hand graphs depict the dynamics within structured metapopulations. Male diversity is maintained both among and very often within subpopulations, and conformity can evolve.

We investigated the verbal model that we developed above using simulations of population dynamics, in order to explore whether conformity can indeed evolve *via* such a mechanism (Figs. 4 and 5). Consider a group of individuals, with the same number of males and females. There are two diallelic genes (or two non‐genetic inclusively heritable variants). The first one, with variants *A* and *B*, is expressed in males only and is neutral for fitness. The second one, with variants *C* and *c*, is expressed in females only: females *c* mate randomly, while females *C* use the logistic conformist learning rule described in Section III.4. without associated cost. The strength of this conformist rule is expressed by the parameter *β*. Initially, the two inclusively heritable types of variants are neither physically nor statistically linked so that they vary independently from each other. Each female has two offspring, one male and one female that inherit the traits from their parents.

The metapopulation is composed of several such groups. In each new generation, a random fraction *d* of the offspring migrates to another randomly chosen subpopulation on one of the spots left vacant by other migrants (males take male spots and females take female spots to prevent sex bias in groups). Initially, alleles *A* and *B* are randomly allocated in the meta‐population in 50:50 proportions. Alleles *C* and *c* are also randomly allocated, with an initial proportion *q*0 of allele *C*. We explored the effect of the dispersal rate *d* and of the conformity strength parameter *β* on the proportion of *A* and *B* males and of *C* and *c* females (Fig. 4), taking the initial proportion of *q*0 of conformists and the recombination rate *r* fixed (*q*0 = *r* = 0.2).

Simulations show that conformity can indeed evolve under the sole effect of the Fisher runaway process unfolding independently in every subpopulation (Fig. 4). This happens in a particular region of the parameter space, namely, when the dispersal rate *d* is not too high compared to the conformity strength parameter *β*. A likely explanation is that when the dispersal rate is too high, the spatial structure of the population becomes less relevant. However, more theoretical work is needed to understand the precise role of each parameter in the evolution of conformity *via* the Fisher runaway process.

Our simulation results mirror those of Somveille *et al*. (2018), who study the effect of similar parameters in a computational model of the emergence of local traditions. Similarly, they find that local traditions emerge when the dispersal rate is not too high compared to the strength of conformity. The difference with our model is that they consider conformity as a common feature of all individuals and study the consequences on behaviour adoption. By contrast, we study whether conformity can evolve in the first place through the Fisher runaway process. In our case, local traditions emerge because conformity has been able to evolve. Our goal here is not to illustrate all the properties of this simple model but rather to show that under some parameter values, conformity does evolve under the sole effect of the Fisher runaway process unfolding independently in every subpopulation.

As an illustration, we also show how the population dynamics unfold for a set of parameters for which conformity evolves (Fig. 5) and compare this to the case wherein the population is spatially unstructured. For these parameter values, conformity evolves readily in metapopulations, and leads to among‐group diversity on the male phenotype. When the population is unstructured however (the same number of individuals but no subgroups and therefore no migration), male diversity is quickly lost, and conformity becomes neutral.

## VII. CONCLUSIONS

(1) The strength of our proposed pathway rooted in mate choice and runaway sexual selection is that it explains the evolution of social learning and conformity, as well as culture.

(2) One of the major challenges therefore for empirical studies of social learning is to find out whether the detection of majority behaviour in mate copying preceded (in evolutionary time) the detection of majority behaviour in other contexts such as foraging and to what extent it evolved analogously or homologously. For that goal we will need to determine in a large range of species the shape of the response function of conformity in mate choice, and other contexts with experiments manipulating the level of majority. Altogether, this provides a rich agenda for future research.

(3) In the expectation of such information, the tentative model we propose and simulate here for the evolution of conformity, and all its cultural evolution consequences, casts surprising light on one of the major processes that has participated in making us humans. Sex might play a bigger role than previously thought in the long‐run development of cultural traditions.

## References

[brv12899-bib-0001] Acerbi, A. , van Leeuwen, E. J. , Haun, D. B. & Tennie, C. (2016). Conformity cannot be identified based on population‐level signatures. Scientific Reports 6, 36068. 27796373 10.1038/srep36068 PMC5086853

[brv12899-bib-0002] Anderson, L. R. & Holt, C. A. (1997). Information cascades in the laboratory. The American Economic Review 87, 847–862.

[brv12899-bib-0003] Aoki, K. & Feldman, M. W. (2014). Evolution of learning strategies in temporally and spatially variable environments: a review of theory. Theoretical Population Biology 91, 3–19. 24211681 10.1016/j.tpb.2013.10.004 PMC4412376

[brv12899-bib-0004] Aoki, K. , Lehmann, L. & Feldman, M. W. (2011). Rates of cultural change and patterns of cultural accumulation in stochastic models of social transmission. Theoretical Population Biology 79, 192–202. 21315753 10.1016/j.tpb.2011.02.001

[brv12899-bib-0005] Aplin, L. M. , Farine, D. R. , Morand‐Ferron, J. , Cockburn, A. , Thornton, A. & Sheldon, B. C. (2015 *a*). Counting conformity: evaluating the units of measurement in frequency‐dependent social learning. Animal Behaviour 110, e5–e8.

[brv12899-bib-0006] Aplin, L. M. , Farine, D. R. , Morand‐Ferron, J. , Cockburn, A. , Thornton, A. & Sheldon, B. C. (2015 *b*). Experimentally induced innovations lead to persistent culture via conformity in wild birds. Nature 518, 538–541. 25470065 10.1038/nature13998 PMC4344839

[brv12899-bib-0007] Aplin, L. M. , Sheldon, B. C. & McElreath, R. (2017). Conformity does not perpetuate suboptimal traditions in a wild population of songbirds. Proceedings of the National Academy of Sciences 114, 7830–7837. 10.1073/pnas.1621067114 PMC5544276 28739943

[brv12899-bib-0008] Asch, S. E. (1955). Opinions and social pressure. Scientific American 193, 31–35.

[brv12899-bib-0009] Ayoub, R. , Armstrong, E. & Miller, N. Y. (2019). Out of sight, out of mind: mechanisms of social choice in fish. Animal Behaviour 155, 163–169.

[brv12899-bib-0010] Bailey, N. W. & Moore, A. J. (2012). Runaway sexual selection without genetic correlations: social environments and flexible mate choice initiate and enhance the Fisher process. Evolution 66, 2674–2684. 22946795 10.1111/j.1558-5646.2012.01647.x PMC3627302

[brv12899-bib-0011] Banerjee, A. V. (1992). A simple model of herd behavior. The Quarterly Journal of Economics 107, 797–817.

[brv12899-bib-0012] Baron, R. S. , Vandello, J. A. & Brunsman, B. (1996). The forgotten variable in conformity research: impact of task importance on social influence. Journal of Personality and Social Psychology 71, 915–927.

[brv12899-bib-0013] Barta, Z. & Giraldeau, L. A. (2001). Breeding colonies as information centers: a reappraisal of information‐based hypotheses using the producer‐scrounger game. Behavioral Ecology 12, 121–127.

[brv12899-bib-0014] Battesti, M. , Moreno, C. , Joly, D. & Mery, F. (2015). Biased social transmission in *Drosophila* oviposition choice. Behavioral Ecology and Sociobiology 69, 83–87.

[brv12899-bib-0015] Bernheim, D. (1994). A theory of conformity. Journal of Political Economy 102, 841–877.

[brv12899-bib-0016] Bikhchandani, S. , Hirshleifer, D. & Welch, I. (1992). A theory of fads, fashion, custom, and cultural change as informational cascades. Journal of Political Economy 100, 992–1026.

[brv12899-bib-0017] Bond, R. (2005). Group size and conformity. Group Processes & Intergroup Relations 8, 331–354.

[brv12899-bib-0018] Bond, R. & Smith, P. B. (1996). Culture and conformity: a meta‐analysis of studies using Asch's (1952b, 1956) line judgment task. Psychological Bulletin 119, 111–137.

[brv12899-bib-0019] Boulinier, T. , Yoccoz, N. G. , McCoy, K. D. , Erikstad, K. E. & Tveraa, T. (2002). Testing the effect of conspecific reproductive success on dispersal and recruitment decisions in a colonial bird: design issues. Journal of Applied Statistics 29, 509–520.

[brv12899-bib-0020] Boyd, R. & Richerson, P. J. (1985). Culture and the Evolutionary Process. University of Chicago Press, Chicago, IL.

[brv12899-bib-0021] Boyd, R. & Richerson, P. J. (2005). The Origin and Evolution of Cultures. Oxford University Press, Oxford.

[brv12899-bib-0022] Brown, C. & Irving, E. (2014). Irving, individual personality traits influence group exploration in a feral guppy population. Behavioral Ecology 25, 95–101.

[brv12899-bib-0023] Brown, C. & Laland, K. N. (2002). Social learning of a novel avoidance task in the guppy: conformity and social release. Animal Behaviour 64, 41–47.

[brv12899-bib-0024] Brown, C. R. & Bomberger Brown, M. (2001). Avian coloniality. Progress and problems. In Current Ornithology (eds J. Val Nolan and C. F. Thompson ), pp. 1–81. Kluvwer Academic/Plenum Publishers, New York.

[brv12899-bib-0025] Buckley, N. J. (1997). Spatial‐concentration effects and the importance of local enhancement in the evolution of colonial breeding in seabirds. American Naturalist 149, 1091–1112. 10.1086/286040 18811265

[brv12899-bib-0026] Bulow, J. , Geanakoplos, J. & Klemperer, P. (1985). Multimarket oligopoly: strategic substitutes and strategic complements. Journal of Political Economy 93, 488–511.

[brv12899-bib-0027] Burdett, E. R. , Lucas, D. , Buchsbaum, D. , McGuian, N. , Wood, L. A. & Whiten, A. (2016). Do children copy an expert or a majority? Examining selective learning in instrumental and normative contexts. PLoS One 11, e0164698. 27768716 10.1371/journal.pone.0164698 PMC5074571

[brv12899-bib-0028] Cantor, M. , Shoemaker, L. G. , Cabral, R. B. , Flores, C. O. , Varga, M. & Whitehead, H. (2015). Multilevel animal societies can emerge from cultural transmission. Nature Communications 6, 8091. 10.1038/ncomms9091 PMC4569709 26348688

[brv12899-bib-0029] Cantor, M. & Whitehead, H. (2013). The interplay between social networks and culture: theoretically and among whales and dolphins. Philosophical Transactions of the Royal Society B 368, 20120340. 10.1098/rstb.2012.0340 PMC3638443 23569288

[brv12899-bib-0030] Cavalli‐Sforza, L. L. & Feldman, M. W. (1981). Cultural Transmission and Evolution: A Quantitative Approach. Princeton University Press, Princeton, NJ. 7300842

[brv12899-bib-0031] Cherng, C. G. , Wang, C. Y. , Lai, Y. T. , Tzeng, W. Y. , Chen, L. H. , Tsai, Y. N. & Yu, L. (2014). Anticipated, intense risk enhances behavioral conformity in a mouse model. Ethology 120, 1035–1043.

[brv12899-bib-0032] Cialdini, R. B. & Goldstein, N. J. (2004). Social influence: compliance and conformity. Annual Review of Psychology 55, 591–621. 10.1146/annurev.psych.55.090902.142015 14744228

[brv12899-bib-0033] Claidière, N. & Whiten, A. (2012). Integrating the study of conformity and culture in humans and non‐human animals. Psychological Bulletin 138, 126–145. 22061691 10.1037/a0025868

[brv12899-bib-0034] Coleman, S. (2004). The effect of social conformity on collective voting behavior. Political Analysis 12, 76–96.

[brv12899-bib-0035] Crast, J. , Hardy, J. M. & Fragaszy, D. (2010). Inducing traditions in captive capuchin monkeys (*Cebus apella*). Animal Behaviour 80, 955–964.

[brv12899-bib-0036] Cresswell, W. & Quinn, J. L. (2011). Predicting the optimal prey group size from predator hunting behaviour. Journal of Animal Ecology 80, 310–319. 21244418 10.1111/j.1365-2656.2010.01775.x

[brv12899-bib-0037] Danchin, É. , Boulinier, T. & Massot, M. (1998). Conspecific reproductive success and breeding habitat selection: implications for the study of coloniality. Ecology 79, 2415–2428.

[brv12899-bib-0038] Danchin, É. , Charmantier, A. , Champagne, F. , Mesoudi, A. , Pujol, B. & Blanchet, S. (2011). Beyond DNA: integrating inclusive inheritance into an extended theory of evolution. Nature Reviews Genetics 12, 475–486. 10.1038/nrg3028 21681209

[brv12899-bib-0039] Danchin, E. , Giraldeau, L. A. & Cézilly, F. (2008). Behavioral Ecology. Oxford University Press, London.

[brv12899-bib-0040] Danchin, É. , Nöbel, S. , Pocheville, A. , Dagaeff, A.‐C. , Demay, L. , Alphand, M. , Ranty‐Roby, S. , van Renssen, L. , Monier, M. , Gazagne, E. , Allain, M. & Isabel, G. (2018). Cultural flies: conformist social learning in fruitflies predicts long‐lasting mate‐choice traditions. Science 362, 1025–1030. 30498121 10.1126/science.aat1590

[brv12899-bib-0041] Danchin, É. & Wagner, R. H. (1997). The evolution of coloniality: the emergence of new perspectives. Trends in Ecology and Evolution 12, 342–347. 21238100 10.1016/s0169-5347(97)01124-5

[brv12899-bib-0042] Danchin, É. & Wagner, R. H. (2010). Inclusive heritability: combining genetic and non‐genetic information to study animal behavior and culture. Oikos 119, 210–218.

[brv12899-bib-0043] David, B. & Turner, J. C. (1996). Studies in self categorization and minority conversion: is being a member of the out‐group an advantage? British Journal of Social Psychology 35, 179–199.

[brv12899-bib-0044] Davies, N. B. & Krebs, J. R. (1984). Behavioral Ecology. Blackwell Scientific, Oxford.

[brv12899-bib-0045] Day, R. L. , MacDonald, T. , Brown, C. , Laland, K. N. & Reader, S. M. (2001). Interactions between shoal size and conformity in guppy social foraging. Animal Behaviour 62, 917–925.

[brv12899-bib-0046] Denton, K. K. , Ram, Y. , Liberman, U. & Feldman, M. W. (2020). Cultural evolution of conformity and anticonformity. Proceedings of the National Academy of Sciences 117, 13603–13614. 10.1073/pnas.2004102117 PMC7306811 32461360

[brv12899-bib-0047] Deutsch, M. & Gerard, H. B. (1955). A study of normative and informational social influences upon individual judgment. The Journal of Abnormal and Social Psychology 51, 629–636. 10.1037/h0046408 13286010

[brv12899-bib-0048] DeYoung, C. G. , Peterson, J. B. & Higgins, D. M. (2002). Higher‐order factors of the big five predict conformity: are there neuroses of health? Personality and Individual Differences 33, 533–552.

[brv12899-bib-0049] Dindo, M. , Whiten, A. & de Waal, F. B. (2009). In‐group conformity sustains different foraging traditions in capuchin monkeys (*Cebus apella*). PLoS One 4, e7858. 19924242 10.1371/journal.pone.0007858 PMC2773420

[brv12899-bib-0050] Doligez, B. , Cadet, C. , Danchin, E. & Boulinier, T. (2003). When to use public information for breeding habitat selection? The role of environmental predictability and density dependence. Animal Behaviour 66, 973–988.

[brv12899-bib-0051] Doligez, B. , Danchin, E. & Clobert, J. (2002). Public information and breeding habitat selection in a wild bird population. Science 297, 1168–1170. 12183627 10.1126/science.1072838

[brv12899-bib-0052] Doligez, B. , Danchin, É. , Clobert, J. & Gustafsson, L. (1999). The use of conspecific reproductive success for breeding habitat selection in a non‐colonial, hole‐nesting species, the collared flycatcher. Journal of Animal Ecology 68, 1193–1206.

[brv12899-bib-0053] Dukas, R. & Edelstein‐Keshet, L. (1998). The spatial distribution of colonial food provisioners. Journal of Theoretical Biology 190, 121–134.

[brv12899-bib-0054] Eberhard, J. R. (2002). Cavity adoption and the evolution of coloniality in cavity‐nesting birds. The Condor 104, 240–247.

[brv12899-bib-0055] Efferson, C. , Lalive, R. , Cacult, M. P. & Kistler, D. (2016). The evolution of facultative conformity based on similarity. PLoS One 11, e0168551. 28002461 10.1371/journal.pone.0168551 PMC5176289

[brv12899-bib-0056] Efferson, C. , Lalive, R. , Richerson, P. J. , Mcelreath, R. & Lubell, M. (2008). Conformists and mavericks: the empirics of frequency‐dependent cultural transmission. Evolution and Human Behavior 29, 56–64.

[brv12899-bib-0057] Enquist, M. , Eriksson, K. & Ghirlanda, S. (2007). Critical social learning: a solution to Rogers's paradox of nonadaptive culture. American Anthropologist 109, 727–734.

[brv12899-bib-0058] Feldman, M. W. , Aoki, K. & Kumm, J. (1996). Individual versus social learning: evolutionary analysis in a fluctuating environment. Anthropological Science 104, 209–231.

[brv12899-bib-0059] Fisher, R. A. (1930). The Genetical Theory of Natural Selection. Clarendon Press, Oxford, UK.

[brv12899-bib-0060] Franz, M. & Matthews, L. J. (2010). Social enhancement can create adaptive, arbitrary and maladaptive cultural traditions. Proceedings of the Royal Society B: Biological Sciences 277, 3363–3372. 10.1098/rspb.2010.0705 PMC2981925 20547762

[brv12899-bib-0061] Fürstbauer, I. & Fry, A. (2018). Social conformity in solitary crabs, *Carcinus maenas*, is driven by individual differences in behavioural plasticity. Animal Behaviour 135, 131–137.

[brv12899-bib-0062] Gelblum, A. , Pinkoviezky, I. , Fonio, E. , Ghosh, A. , Gov, N. & Feinerman, O. (2015). Ant groups optimally amplify the effect of transiently informed individuals. Nature Communications 6, 7729. 10.1038/ncomms8729 PMC4525283 26218613

[brv12899-bib-0063] Griskevicius, V. , Goldstein, N. J. , Mortensen, C. R. , Cialdini, R. B. & Kenrick, D. T. (2006). Going along versus going alone: when fundamental motives facilitate strategic (non) conformity. Journal of Personality and Social Psychology 91, 281–294. 16881765 10.1037/0022-3514.91.2.281

[brv12899-bib-0064] Gúzman, R. A. , Rodríguez‐Sickert, C. & Rowthorn, R. (2007). When in Rome, do as the Romans do: the coevolution of altruistic punishment, conformist learning, and cooperation. Evolution and Human Behavior 28, 112–117.

[brv12899-bib-0065] Haun, D. B. , Rekers, Y. & Tomasello, M. (2012). Majority‐biased transmission in chimpanzees and human children, but not orangutans. Current Biology 22, 727–731. 22503497 10.1016/j.cub.2012.03.006

[brv12899-bib-0066] Haun, D. B. , Rekers, Y. & Tomasello, M. (2014). Children conform to the behavior of peers; other great apes stick with what they know. Psychological Science 25, 2160–2167. 25355648 10.1177/0956797614553235

[brv12899-bib-0067] Haun, D. B. M. , van Leeuwen, E. J. C. & Edelson, M. G. (2013). Majority influence in children and other animals. Developmental Cognitive Neuroscience 3, 61–71. 23245221 10.1016/j.dcn.2012.09.003 PMC6987688

[brv12899-bib-0068] Hellström, G. , Heynen, M. , Oosten, J. , Borcherding, J. & Magnhagen, C. (2011). The effect of group size on risk taking and social conformity in Eurasian perch. Ecology of Freshwater Fish 20, 499–502.

[brv12899-bib-0069] Henrich, J. & Boyd, R. (1998). The evolution of conformist transmission and the emergence of between‐group differences. Evolution and Human Behavior 19, 215–241.

[brv12899-bib-0070] Henrich, J. & Boyd, R. (2001). Why people punish defectors: weak conformist transmission can stabilize costly enforcement of norms in cooperative dilemmas. Journal of Theoretical Biology 208, 79–89. 11162054 10.1006/jtbi.2000.2202

[brv12899-bib-0071] Henrich, J. & McElreath, R. (2003). The evolution of cultural evolution. Evolutionary Anthropology 12, 123–135.

[brv12899-bib-0072] Herbert‐Read, J. E. , Krause, S. , Morrell, L. J. , Schaerf, T. M. , Krause, J. & Ward, A. J. W. (2013). The role of individuality in collective group movement. Proceedings of the Royal Society B: Biological Science 280, 20122564. 10.1098/rspb.2012.2564 PMC3574314 23222452

[brv12899-bib-0073] Heyes, C. M. (1994). Social learning in animals: categories and mechanisms. Biological Reviews 69, 207–231. 8054445 10.1111/j.1469-185x.1994.tb01506.x

[brv12899-bib-0074] Hopper, L. M. , Shapiro, S. J. , Lambeth, S. P. & Brosnan, F. (2011). Chimpanzees' socially maintained food preferences indicate both conservatism and conformity. Animal Behaviour 81, 1195–1202. 27011390 10.1016/j.anbehav.2011.03.002 PMC4801479

[brv12899-bib-0075] Jenness, A. (1932). The role of discussion in changing opinion regarding a matter of fact. Journal of Personality and Social Psychology 27, 279–296.

[brv12899-bib-0076] Jolles, J. W. , de Visser, L. & van den Bos, R. (2011). Male Wistar rats show individual differences in an animal model of conformity. Animal Cognition 14, 769–773. 21465219 10.1007/s10071-011-0395-4 PMC3162626

[brv12899-bib-0077] Kendal, J. , Giraldeau, L. A. & Laland, K. (2009). The evolution of social learning rules: payoff‐biased and frequency‐dependent biased transmission. Journal of Theoretical Biology 260, 210–219. 19501102 10.1016/j.jtbi.2009.05.029

[brv12899-bib-0078] King, A. J. , Williams, L. J. & Mettke‐Hofmann, C. (2015). The effects of social conformity on Gouldian finch personality. Animal Behaviour 99, 25–31.

[brv12899-bib-0079] Koski, E. & Burkart, J. M. (2015). Common marmosets show social plasticity and group‐level similarity in personality. Scientific Reports 5, 8878. 25743581 10.1038/srep08878 PMC5155412

[brv12899-bib-0080] Kuran, T. & Sandholm, W. H. (2008). Cultural integration and its discontents. Review of Economic Studies 75, 201–228.

[brv12899-bib-0081] Lachlan, R. F. , Ratmann, O. & Nowicki, S. (2018). Cultural conformity generates extremely stable traditions in bird song. Nature Communications 9, 2417. 10.1038/s41467-018-04728-1 PMC6010409 29925831

[brv12899-bib-0082] Laland, K. N. (1994). Sexual selection with a culturally transmitted mating preference. Theoretical Population Biology 45, 1–15. 8023313 10.1006/tpbi.1994.1001

[brv12899-bib-0083] Laland, K. N. (2004). Social learning strategies. Animal Learning & Behavior 32, 4–14. 10.3758/bf03196002 15161136

[brv12899-bib-0084] Laland, K. N. , Atton, N. & Webster, M. M. (2011). From fish to fashion: experimental and theoretical insights into the evolution of culture. Philosophical Transactions of the Royal Society B 366, 958–968. 10.1098/rstb.2010.0328 PMC3049094 21357218

[brv12899-bib-0085] Latané, B. (1981). The psychology of social impact. American Psychologist 36, 343–356.

[brv12899-bib-0086] Latané, B. & Wolf, S. (1981). The social impact of majorities and minorities. Psychological Review 88, 438–453.

[brv12899-bib-0087] Leca, J. B. , Gunst, N. & Huffman, M. A. (2010). Indirect social influence in the maintenance of the stone‐handling tradition in Japanese macaques, *Macaca fuscata* . Animal Behaviour 79, 117–126.

[brv12899-bib-0088] Lecheval, V. , Jiang, L. , Tichit, P. , Sire, C. , Hemelrijk, C. K. & Theraulaz, G. (2018). Social conformity and propagation of information in collective U‐turns of fish schools. Proceedings of the Royal Society B: Biological Sciences 285, 20180251. 10.1098/rspb.2018.0251 PMC5936730 29695447

[brv12899-bib-0089] Lehmann, L. & Feldman, M. V. (2008). The co‐evolution of culturally inherited altruistic helping and cultural transmission under random group formation. Theoretical Population Biology 73, 506–516. 18420241 10.1016/j.tpb.2008.02.004

[brv12899-bib-0090] Luncz, L. V. & Boesch, C. (2014). Tradition over trend: neighboring chimpanzee communities maintain differences in cultural behavior despite frequent immigration of adult females. American Journal of Primatology 76, 649–657. 24482055 10.1002/ajp.22259

[brv12899-bib-0091] Magnhagen, C. (2012). Personalities in a crowd: what shapes the behaviour of Eurasian perch and other shoaling fishes? Current Zoology 58, 35–44.

[brv12899-bib-0092] McDonald, N. D. , Rands, S. A. , Hill, F. , Elder, C. C. & Ioannou, C. C. (2016). Consensus and experience trump leadership, suppressing individual personality during social foraging. Science Advances 2, e1600892. 27652342 10.1126/sciadv.1600892 PMC5023318

[brv12899-bib-0093] McFadden, D. (1973). Conditional logit analysis of qualitative choice behavior. In Frontiers in Econometrics (ed. P. Zamremba ). Academic Press, New York.

[brv12899-bib-0094] Mengel, F. (2009). Conformism and cooperation in a local interaction model. Journal of Evolutionary Economics 19, 397–415.

[brv12899-bib-0095] Merrell, F. (2011). Conformity and resistance as cultural process in postmodern globalizing times. Semiotica 183, 77–104.

[brv12899-bib-0096] Mesoudi, A. (2018). Migration, acculturation, and the maintenance of between‐group cultural variation. PLoS One 13, e0205573. 30325943 10.1371/journal.pone.0205573 PMC6191118

[brv12899-bib-0097] Molleman, L. , Pen, I. & Weissing, F. J. (2013 *a*). Effects of conformism on the cultural evolution of social behaviour. PLoS One 8, e68153. 23874528 10.1371/journal.pone.0068153 PMC3707918

[brv12899-bib-0098] Molleman, L. , Quinones, A. E. & Weissing, F. J. (2013 *b*). Cultural evolution of cooperation: the interplay between forms of social learning and group selection. Evolution and Human Behavior 34, 342–349.

[brv12899-bib-0099] Morgan, T. H. J. , Acerbi, A. & van Leeuwen, E. J. C. (2019). Copy‐the‐majority of instances or individuals? Two approaches to the majority and their consequences for conformist decision‐making. PLoS One 14, e0210748. 30682728 10.1371/journal.pone.0210748 PMC6347471

[brv12899-bib-0100] Morgan, T. J. H. & Laland, K. N. (2012). The biological bases of conformity. Frontiers in Neuroscience 6, 87. 22712006 10.3389/fnins.2012.00087 PMC3375089

[brv12899-bib-0101] Muthukrishna, M. , Morgan, T. J. & Henrich, J. (2016). The when and who of social learning and conformist transmission. Evolution and Human Behavior 37, 10–20.

[brv12899-bib-0102] Nakahashi, W. (2007). The evolution of conformist transmission in social learning when the environment changes periodically. Theoretical Population Biology 72, 52–66. 17442355 10.1016/j.tpb.2007.03.003

[brv12899-bib-0103] Nakahashi, W. , Wakano, J. Y. & Henrich, J. (2012). Adaptive social learning strategies in temporally and spatially varying environments. Human Nature 23, 386–418. 22926986 10.1007/s12110-012-9151-y

[brv12899-bib-0104] Nelson, D. A. & Poesel, A. (2009). Does learning produce song conformity or novelty in white‐crowned sparrows, *Zonotrichia leucophrys*? Animal Behaviour 78, 433–440.

[brv12899-bib-0105] Nelson, D. A. & Poesel, A. (2014). Tutor choice and imitation accuracy during song learning in a wild population of the Puget Sound white‐crowned sparrow. Behavioral Ecology and Sociobiology 68, 1741–1752.

[brv12899-bib-0106] Parejo, D. , White, J. F. , Clobert, J. , Dreiss, A. N. & Danchin, E. (2007). Blue tits use fledging quantity and quality as public information in breeding habitat choice. Ecology 88, 2373–2382. 17918414 10.1890/06-2000.1

[brv12899-bib-0107] Peña, J. , Volken, H. , Pestelacci, E. & Tomassini, M. (2009). Conformity hinders the evolution of cooperation on scale‐free networks. Physical Review E 80, 016110. 10.1103/PhysRevE.80.016110 19658777

[brv12899-bib-0108] Perreault, C. , Moya, C. & Boyd, R. (2012). A Bayesian approach to the evolution of social learning. Evolution and Human Behavior 33, 449–459.

[brv12899-bib-0109] Perry, S. (2009). Conformism in the food processing techniques of white‐faced capuchin monkeys (*Cebus capucinus*). Animal Cognition 12, 705–716. 19455357 10.1007/s10071-009-0230-3 PMC2728904

[brv12899-bib-0110] Pike, T. W. & Laland, K. N. (2010). Conformist learning in nine‐spined sticklebacks' foraging decisions. Biology Letters 6, 466–468. 20129948 10.1098/rsbl.2009.1014 PMC2936200

[brv12899-bib-0111] Raihani, N. J. , Thornton, A. & Bshary, R. (2012). Punishment and cooperation in nature. Trends in Ecology & Evolution 27, 288–295. 22284810 10.1016/j.tree.2011.12.004

[brv12899-bib-0112] Rendell, L. , Forgarty, L. , Hoppitt, W. J. , Morgan, T. J. , Webster, M. M. & Laland, K. N. (2011). Cognitive culture: theoretical and empirical insights into social learning strategies. Trends in Cognitive Science 15, 68–76. 10.1016/j.tics.2010.12.002 21215677

[brv12899-bib-0113] Rolland, C. , Danchin, É. & de Fraipont, M. (1998). The evolution of coloniality in birds in relation to food, habitat, predation, and life‐history traits: a comparative analysis. American Naturalist 151, 514–529. 10.1086/286137 18811373

[brv12899-bib-0114] Schnoell, A. V. & Fichtel, C. (2012). Wild redfronted lemurs (*Eulemur rufifrons*) use social information to learn new foraging techniques. Animal Cognition 15, 505–516. 22426747 10.1007/s10071-012-0477-y PMC3377903

[brv12899-bib-0115] Serrano, D. , Tella, J. L. , Forero, M. G. & Donazar, J. A. (2001). Factors affecting breeding dispersal in the facultatively colonial lesser kestrel: individual experience vs. conspecific cues. Journal of Animal Ecology 70, 568–578.

[brv12899-bib-0116] Sherif, M. (1935). A study of some social factors in perception. Archives of Psychology 187, 60.

[brv12899-bib-0117] Siegel‐Causey, D. & Kharitonov, S. P. (1990). The evolution of coloniality. In Current Ornithology (ed. D. M. Power ), pp. 285–330. Plenum Press, New York.

[brv12899-bib-0118] Singh, M. & Boomsma, J. J. (2015). Policing and punishment across the domains of social evolution. Oikos 124, 971–982.

[brv12899-bib-0119] Smaldino, P. E. , Aplin, L. M. & Farine, D. R. (2018). Sigmoidal acquisition curves are good indicators of conformist transmission. Scientific Reports 8, 14015. 30228351 10.1038/s41598-018-30248-5 PMC6143626

[brv12899-bib-0120] Somveille, M. , Firth, J. A. , Aplin, L. M. , Farine, D. R. , Sheldon, B. C. & Thompson, R. N. (2018). Movement and conformity interact to establish local behavioural traditions in animal populations. PLoS Computational Biology 14, e1006647. 30571696 10.1371/journal.pcbi.1006647 PMC6319775

[brv12899-bib-0121] Sterelny, K. (2006). The evolution and evolvability of culture. Mind & Language 21, 137–165.

[brv12899-bib-0122] Sumpter, D. J. T. & Beekman, M. (2003). From nonlinearity to optimality: pheromone trail foraging by ants. Animal Behaviour 66, 273–280.

[brv12899-bib-0123] van Cleve, J. (2016). Cooperation, conformity, and the coevolutionary problem of trait associations. Journal of Theoretical Biology 396, 13–24. 26907203 10.1016/j.jtbi.2016.02.012

[brv12899-bib-0124] van de Waal, E. , Borgeaud, C. & Whiten, A. (2013). Potent social learning and conformity shape a wild primate's foraging decisions. Science 340, 483–485. 23620053 10.1126/science.1232769

[brv12899-bib-0125] van Leeuwen, E. J. & Haun, D. (2013). Conformity in primates: fad or fact? Evolution and Human Behavior 34, 1–7.

[brv12899-bib-0126] van Leeuwen, E. J. , Kendal, R. L. , Tennie, C. & Haun, D. (2015). Conformity and its look‐a‐likes. Animal Behaviour 110, e1–e4.

[brv12899-bib-0127] Varela, S. A. M. , Danchin, É. & Wagner, R. H. (2007). Does predation select for or against avian coloniality? A comparative analysis. Journal of Evolutionary Biology 20, 1490–1503. 17584242 10.1111/j.1420-9101.2007.01334.x

[brv12899-bib-0128] Varela, S. A. M. , Matos, M. & Schlupp, I. (2018). The role of mate‐choice copying in speciation and hybridization. Biological Reviews 93, 1304–1322. 29417719 10.1111/brv.12397

[brv12899-bib-0129] Wakano, J. Y. & Aoki, K. (2007). Do social learning and conformist bias coevolve? Henrich and Boyd revisited. Theoretical Population Biology 72, 504–512. 17561216 10.1016/j.tpb.2007.04.003

[brv12899-bib-0130] Watson, S. K. , Lambeth, S. P. , Schapiro, S. J. & Whiten, A. (2018). Chimpanzees prioritise social information over pre‐existing behaviours in a group context but not in dyads. Animal Cognition 21, 407–418. 29574554 10.1007/s10071-018-1178-y PMC5908815

[brv12899-bib-0131] Webster, M. M. & Hart, P. J. (2006). Subhabitat selection by foraging threespine stickleback (*Gasterosteus aculeatus*): previous experience and social conformity. Behavioral Ecology and Sociobiology 60, 77–86.

[brv12899-bib-0132] Whitehead, H. & Richerson, P. J. (2009). The evolution of conformist social learning can cause population collapse in realistically variable environments. Evolution and Human Behavior 30, 261–273.

[brv12899-bib-0133] Whiten, A. (2019). Conformity and over‐imitation: an integrative review of variant forms of hyper‐reliance on social learning. Advances in the Study of Behavior 51, 31–75.

[brv12899-bib-0134] Whiten, A. (2021). The burgeoning reach of animal culture. Science 372, eabe6514. 33795431 10.1126/science.abe6514

[brv12899-bib-0135] Whiten, A. , Horner, V. & de Waal, F. B. (2005). Conformity to cultural norms of tool use in chimpanzees. Nature 437, 737–740. 16113685 10.1038/nature04047

[brv12899-bib-0136] Whiten, A. , Spiteri, A. , Horner, V. , Bonnie, K. E. , Lambeth, S. P. , Schapiro, S. J. & De Waal, F. B. (2007). Transmission of multiple traditions within and between chimpanzee groups. Current Biology 17, 1038–1043. 17555968 10.1016/j.cub.2007.05.031

[brv12899-bib-0137] Zala, S. M. , Määttänen, I. & Penn, D. J. (2012). Different social‐learning strategies in wild and domesticated zebrafish, *Danio rerio* . Animal Behaviour 83, 1519–1525.

